# Cryo-EM structure of ALC1 in an open conformation bound to a PARylated nucleosome

**DOI:** 10.1107/S2059798326004158

**Published:** 2026-05-20

**Authors:** Hannah R. Bridges, Luka Bacic, Sebastian Deindl, Guillaume Gaullier

**Affiliations:** a Structura Biotechnology Inc., Toronto, Ontario, Canada; b Division of Cancer Research, Department of Thoracic Surgery, Center for Translational Cell Research (ZTZ), Breisacher Strasse 115, 79106Freiburg, Germany; c Department of Cell and Molecular Biology, Science for Life Laboratory Uppsala University 75124Uppsala Sweden; d Department of Chemistry – Ångström Laboratory Uppsala University 75120Uppsala Sweden; University of Western Australia, Crawley, Australia

**Keywords:** DNA-damage response, chromatin remodeling, ALC1, nucleosome, cryo-EM

## Abstract

Nucleosomes are the repeating unit of chromatin. They act as recognition platforms for chromatin-binding factors that coordinate genome maintenance. The chromatin remodeler Amplified in Liver Cancer 1 (ALC1) is a key component of the DNA-damage response and a promising therapeutic target in cancer. Through extensive classification of our previously deposited cryo-electron microscopy dataset, we identified a previously unresolved ALC1–nucleosome complex characterized by a more open conformation of ALC1. This is the first structure of ALC1 in which all domains are visualized in the context of a nucleosome complex, including the regulatory macro domain and a single α-helix motif within the linker. This newly identified conformation may represent an intermediate between the auto-inhibited and active states, and provides new structural insights into the conformational transitions that regulate the activity of ALC1.

## Abstract 2

Reanalysis of a publicly available cryo-EM dataset identifies a new conformation of the oncogenic chromatin remodeler ALC1/CHD1L bound to a PARylated nucleosome. This new structure reveals the position of the macro domain in the complex, and may help to understand the large conformational change leading to the activation of ALC1.

## 1. Introduction

### 1.1. Structure and molecular recognition of nucleosomes

**Figure 1:**
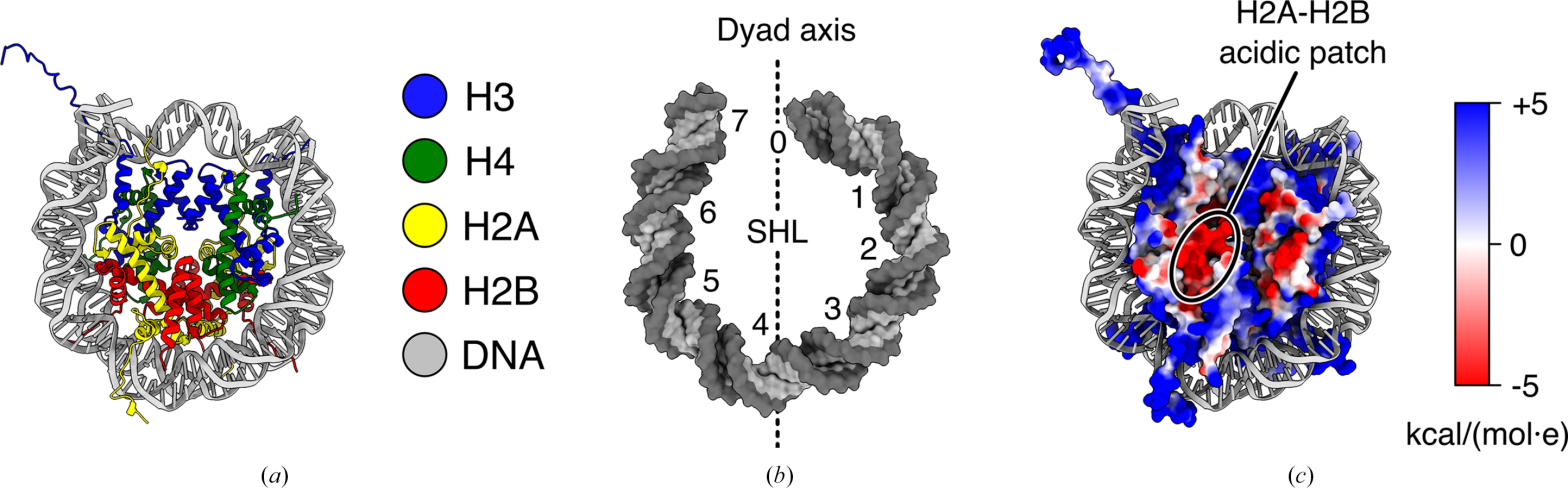
Structure of the nucleosome (PDB entry 1aoi). (*a*) Overview of the nucleosome (disk view). H3 is colored blue, H4 green, H2A yellow, H2B red and DNA gray. (*b*) Superhelical locations (SHLs) are defined as the points where the major groove faces inwards, towards the histone octamer. The base pair at the center of the histone-octamer footprint on the DNA is called the dyad and is numbered bp 0/SHL 0 by convention. Each gyre has seven SHLs, numbered positively on the front gyre and negatively on the back gyre (SHLs with the same numbers but opposite signs refer to equivalent locations related by the pseudo-twofold symmetry of the dyad axis, and a fractional SHL such as 1.5 refers to the minor groove between two SHLs, in this example between SHLs 1 and 2). Only the front gyre is displayed here for clarity. (*c*) Electrostatic potential of the histone octamer. The H2A–H2B acidic patch is labeled.

Eukaryotes package their genomes in a protein–DNA complex called chromatin. The repeating unit of chromatin is the nucleosome (Kornberg, 1974 ▸), which is composed of two copies each of histones H3, H4, H2A and H2B assembled as a histone octamer, around which DNA wraps as almost two turns of a left-handed super-helix (Figs. 1 ▸*a* and 1 ▸*b*; Luger *et al.*, 1997 ▸). In addition to packaging the DNA, nucleosomes provide a recognition platform for chromatin-binding factors involved in a variety of genome-maintenance processes. These proteins recognize specific epitopes on the nucleosome: the H2A–H2B acidic patch (Fig. 1 ▸*c*), the N-terminal tail of H4 or specific post-translational modification (PTM) patterns on histone tails (Zhou *et al.*, 2019 ▸; McGinty & Tan, 2021 ▸). These epitopes are often recognized in combination by chromatin-binding factors harboring multiple ‘reader domains’ within multi-domain proteins or in multi-subunit complexes.

In addition to these canonical epitopes, noncanonical DNA structures such as abasic sites, modified bases, pyrimidine dimers, nicks or double-strand breaks are recognized by DNA damage-sensor proteins (Matsumoto *et al.*, 2019 ▸; Gaullier *et al.*, 2020 ▸; Bilokapic *et al.*, 2020 ▸; Weaver *et al.*, 2022 ▸; Ren *et al.*, 2024 ▸). Furthermore, pioneer transcription factors recognize specific DNA sequences exposed on the outer surface of the nucleosome (Makowski *et al.*, 2020 ▸; Luzete-Monteiro & Zaret, 2022 ▸).

### 1.2. The chromatin remodeler ALC1 in the DNA-damage response

Packaging of the genome into chromatin constitutes a barrier to cellular processes that need physical access to the DNA, such as transcription-factor binding and DNA-damage recognition and repair. To facilitate these processes, eukaryotes have evolved a family of proteins termed ATP-dependent chromatin remodelers (Clapier & Cairns, 2009 ▸; Clapier *et al.*, 2017 ▸). Remodelers can manipulate nucleosomes in a variety of ways, most prominently by performing histone exchange (to install or remove histone variants) or nucleosome translocation by sliding the histone octamer along the DNA (Narlikar *et al.*, 2013 ▸; Bowman & Deindl, 2019 ▸). Like other chromatin-binding factors, remodelers follow general principles of molecular recognition of the nucleosome: they recognize nucleosomal DNA at specific superhelical locations (SHLs; mainly SHL ±2), the N-terminal tail of H4 and often the acidic patch (Markert & Luger, 2021 ▸). Unlike proteins involved purely in recognition and binding, chromatin remodelers undergo large conformational changes during their ATP-hydrolysis cycle. This dynamic nature makes them challenging targets for structure determination: near-atomic resolution structures of remodeler–nucleosome complexes have only become available in the past decade thanks to the advances of the cryo-EM ‘resolution revolution’ (Liu *et al.*, 2017 ▸; Farnung *et al.*, 2017 ▸, 2020 ▸; Ayala *et al.*, 2018 ▸; Eustermann *et al.*, 2018 ▸; Sundaramoorthy *et al.*, 2018 ▸; Willhoft *et al.*, 2018 ▸; Armache *et al.*, 2019 ▸; Chittori *et al.*, 2019 ▸; Li *et al.*, 2019 ▸, 2024 ▸; Patel *et al.*, 2019 ▸; Yan *et al.*, 2019 ▸; Ye *et al.*, 2019 ▸; Han *et al.*, 2020 ▸; He *et al.*, 2020 ▸, 2021 ▸; Wagner *et al.*, 2020 ▸; Baker *et al.*, 2021 ▸; Markert *et al.*, 2021 ▸; Nodelman *et al.*, 2022 ▸, 2025 ▸; Yuan *et al.*, 2022 ▸; Wu *et al.*, 2023 ▸; Zhang *et al.*, 2023 ▸, 2024 ▸; Jalal *et al.*, 2024 ▸; Louder *et al.*, 2024 ▸; Osakabe *et al.*, 2024 ▸; Hu *et al.*, 2025 ▸; James & Farnung, 2025 ▸; Kaur *et al.*, 2025 ▸; Malik *et al.*, 2025 ▸; Sia *et al.*, 2025 ▸; Tian *et al.*, 2025 ▸).

**Figure 2:**
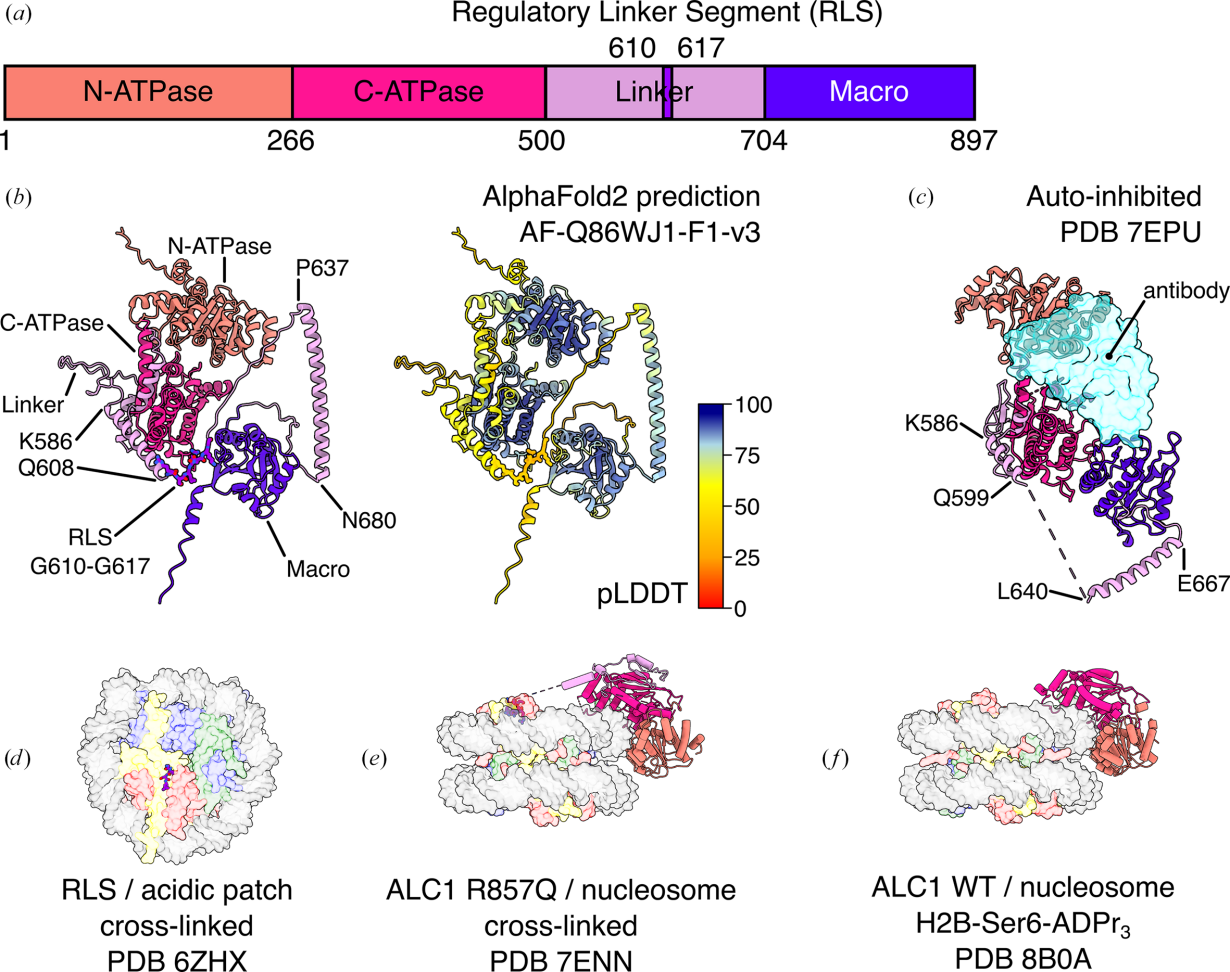
Current structural knowledge about human ALC1. (*a*) Domain structure of ALC1. (*b*) *AlphaFold*2 prediction of full-length ALC1. Left, colored by domain as in (*a*), with domains labeled. Right, colored by pLDDT (prediction confidence). Residues of the regulatory linker segment (RLS) are displayed as sticks. From AlphaFold-DB AF-Q86WJ1-F1-v3. (*c*) Crystal structure of ALC1 in its auto-inhibited state, colored by domain as in (*a*), with the antibody used for crystallization shown as a translucent cyan surface. From PDB entry 7epu. (*d*) Cryo-EM structure of the RLS of ALC1 cross-linked to a nucleosome (disk view). From PDB entry 6zhx. (*e*) Cryo-EM structure of ALC1 R857Q cross-linked to an unmodified nucleosome (gyres view). From PDB entry 7enn. (*f*) Cryo-EM structure of ALC1 bound to a nucleosome site-specifically tri-ADP-ribosylated on H2B-Ser6 (gyres view). From PDB entry 8b0a. In (*d*), (*e*) and (*f*), ALC1 is colored by domain as in (*a*) and the nucleosome is colored as in Fig. 1 ▸(*a*) and shown as a translucent surface for clarity.

Amplified in Liver Cancer 1 (ALC1) is found in vertebrates. Human ALC1 is a 101 kDa protein comprising the N- and C-ATPase domains characteristic of the chromatin-remodeler family, a 200-residue linker and a macro domain unique to this remodeler (Fig. 2 ▸*a*). ALC1 is a key component of the DNA-damage response (Gottschalk *et al.*, 2009 ▸, 2012 ▸; Ahel *et al.*, 2009 ▸). In this cellular pathway, DNA breaks are rapidly recognized by poly-ADP-ribose polymerases 1 and 2 (PARP1 and PARP2; Langelier *et al.*, 2018 ▸). These two enzymes, assisted by their cofactor histone PARylation factor 1 (HPF1), label nucleosomes surrounding a DNA break with poly-ADP-ribose (PAR), a polymeric PTM deposited on histone tails, primarily on Ser10 in H3 and Ser6 in H2B (Leidecker *et al.*, 2016 ▸; Gibbs-Seymour *et al.*, 2016 ▸; Bonfiglio *et al.*, 2017 ▸). ALC1 is rapidly recruited to PARylated chromatin loci, where it catalyzes chromatin relaxation to facilitate the access of downstream DNA-repair factors to the site of DNA damage (Sellou *et al.*, 2016 ▸). This recruitment takes place through a direct physical interaction between PAR chains and the macro domain of ALC1, a PAR reader domain (Karras *et al.*, 2005 ▸). In the absence of PAR, the macro domain interacts with the ATPase domain and maintains ALC1 in an auto-inhibited state. This dual function of the macro domain is critical: its binding to PAR chains serves both as a recruitment mechanism to sites of DNA breaks and as an activation mechanism by displacing the macro domain from the ATPase domain, thereby releasing the auto-inhibition of the remodeler (Lehmann *et al.*, 2017 ▸; Singh *et al.*, 2017 ▸).

### 1.3. Structural information currently available on ALC1

The dynamic nature of chromatin remodelers in general, and the domain structure of ALC1 in particular, with its very long flexible linker between the ATPase and macro domains (Fig. 2 ▸*a*), make structure determination challenging, since all techniques tend to work best with conformationally homogeneous macromolecular complexes. The structure prediction from *AlphaFold*2 is therefore valuable, as it helps in visualizing the segments of ALC1 that are either not captured, or only observed as disconnected fragments, in currently available experimental structures. Interestingly, the linker seems less disordered than had been previously thought: a long α-helix is predicted just upstream of the macro domain (residues Pro637–Asn680) with high confidence (Fig. 2 ▸*b*) and indeed residues Leu640–Glu667 were experimentally confirmed as α-helical in the crystal structure of ALC1 in its auto-inhibited state (Fig. 2 ▸*c*) (Wang *et al.*, 2021 ▸).

We previously identified a regulatory element in the linker of ALC1, demonstrated that it binds to the acidic patch of the nucleosome through an arginine anchor residue (an inter­action mode common among chromatin-binding factors) and determined a cryo-EM structure of this peptide bound and cross-linked to the nucleosome (Fig. 2 ▸*d*; Lehmann *et al.*, 2020 ▸). We refer to this segment of ALC1 as the regulatory linker segment (RLS). *AlphaFold*2 also predicts a helix just upstream of the RLS (residues Lys586–Gln608) with more than 50% confidence (Fig. 2 ▸*b*), which is also partially observed (residues Lys586–Gln599) in the crystal structure of the auto-inhibited state (Fig. 2 ▸*c*; Wang *et al.*, 2021 ▸). This crystal structure was solved with the help of a single-chain variable fragment antibody to stabilize the auto-inhibited conformation and decrease the flexibility of the hinge between the two lobes of the ATPase domain. Neither this strategy nor the crystal packing constrained the more flexible region of the linker, so the RLS is not observed in this structure (Fig. 2 ▸*c*; Wang *et al.*, 2021 ▸). In the same study, this group also determined a cryo-EM structure of the ALC1–nucleosome complex by using the constitutively active ALC1 mutant R857Q in a 5:1 molar excess over unmodified nucleosomes, followed by cross-linking using the *GraFix* protocol (Kastner *et al.*, 2008 ▸). This R857Q mutation, found in some cancers, is located in the macro domain, and abolishes both binding to PAR and auto-inhibition, resulting in unregulated chromatin remodeling by ALC1. The use of this mutant enabled the formation of a complex with an unmodified nucleosome, to which wild-type (WT) ALC1 does not bind. Cross-linking likely stabilized and enabled the visualization of a part of the linker in contact with the C-ATPase lobe and of the interaction between the RLS and the acidic patch (Fig. 2 ▸*e*; Wang *et al.*, 2021 ▸). However, this approach prevented visualization of the macro domain (which is likely to be floating away, only tethered by the end of the linker) and of any loosely bound intermediate states that were not within cross-linking distance and thus lost during purification. At the same time, we were also pursuing a structure of the ALC1–nucleosome complex, but chose a different approach. The new knowledge about the biochemistry of PARP1, PARP2 and HPF1 that developed at the time (Obaji *et al.*, 2018 ▸, 2021 ▸; Gaullier *et al.*, 2020 ▸; Bilokapic *et al.*, 2020 ▸; Suskiewicz *et al.*, 2020 ▸; Sun *et al.*, 2021 ▸; Langelier *et al.*, 2021 ▸) encouraged us to prepare enzymatically PARylated nucleosomes and subsequently form a complex with WT human ALC1. This resulted in a very heterogeneous sample, due to the heterogeneity of PAR chains in sites of attachment, in length and in degree of branching, but enabled the determination of a cryo-EM structure of the natural complex in its active state (Bacic *et al.*, 2021 ▸). Analysis of this cryo-EM dataset with *CryoDRGN* (Zhong *et al.*, 2021 ▸) also hinted at the presence of many more conformational states of ALC1, some clearly more loosely bound to the nucleosome, but time constraints and the image-processing tools available at the time prevented us from further analyzing this complicated heterogeneity. We later used a chemoenzymatic method to produce nucleosomes homogeneously tri-ADP-ribosylated at a single site, Ser6, on a single copy of H2B (Mohapatra *et al.*, 2021 ▸). This approach produced a much more homogeneous ALC1–nucleosome complex and yielded a cryo-EM structure of the active state of ALC1 at higher resolution, but in which the linker and macro domain were still not visualized (Fig. 2 ▸*f*; Bacic *et al.*, 2024 ▸).

Overall, the structural information on ALC1 is therefore still partial and fragmented. Notably, the macro domain has never been observed in the context of an ALC1–nucleosome complex. Due to this gap in structural information, the conformational change caused by the combined recruitment and activation of ALC1 by PARylated nucleosomes has remained unclear.

Here, we revisit our heterogeneous cryo-EM dataset publicly available as EMPIAR-10739 (Bacic *et al.*, 2021 ▸). The sample that yielded this dataset consisted of a nucleosome with a 10 bp DNA arm on one side and no additional DNA on the other side, enzymatically PARylated by PARP2 and HPF1*in vitro*, to which wild-type human ALC1 and ADP–BeF_3_ were subsequently added without trying to purify the PARylated nucleosomes from PARP2 and HPF1. This sample therefore presented a high degree of heterogeneity. We present the structure of a complex with human ALC1 bound to the nucleosome in a more open conformation than that it adopts in the active state, possibly representing an intermediate between the auto-inhibited and active states of the remodeler, and in which all domains of ALC1 can be assigned for the first time in the context of a nucleosome-bound structure.

## 2. Methods

### 2.1. Cryo-EM data processing

**Figure 3:**
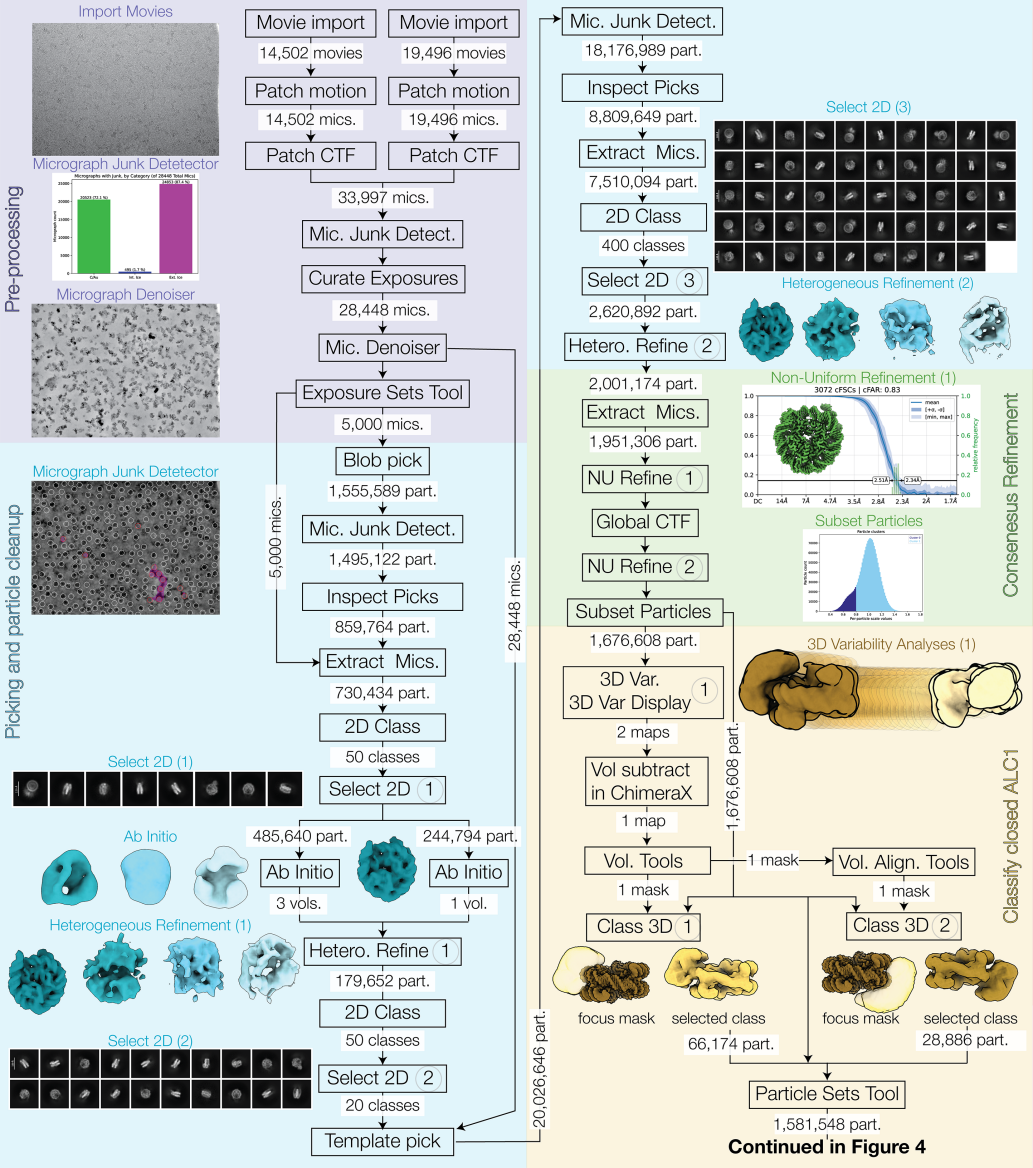
Flow chart of the image-processing workflow (continued in Fig. 4 ▸). The steps of each distinct stage are highlighted in different colors: pre-processing in purple, particle picking and curation in blue, consensus reconstruction in green and classification of the complexes with a closed ALC1 in yellow. Details are provided in Section 2.1, where settings are listed for all steps labeled with a circled number.

**Figure 4:**
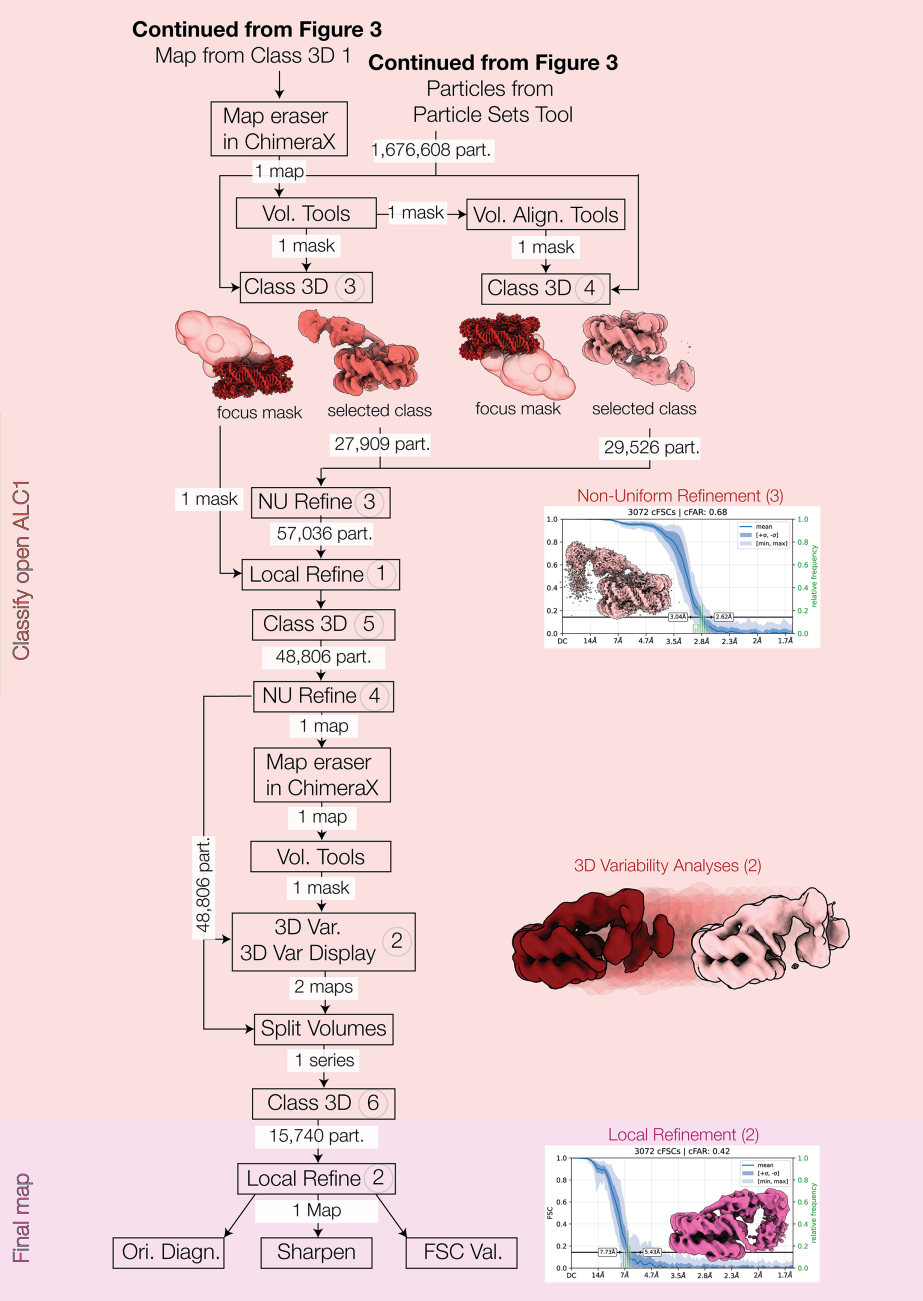
Flow chart of the image-processing workflow (continued from Fig. 3 ▸). The steps of each distinct stage are highlighted in different colors: classification of the complexes with an open ALC1 in red and final refinement of this open complex in pink. Details are provided in Section 2.1, where settings are listed for all steps labeled with a circled number.

All processing steps were conducted using *CryoSPARC* version 4.7.1 (Punjani *et al.*, 2017 ▸) and *ChimeraX* version 1.9 (Pettersen *et al.*, 2021 ▸). Data-collection, image-processing and model-refinement statistics are reported in Supplementary Table S1. Throughout Section 2.1, processing steps designated with numbers in parentheses refer to the corresponding circled numbers in Figs. 3 and 4.

#### 2.1.1. Pre-processing

Movies from EMPIAR-10739 were imported into *Cryo­SPARC* as two separate jobs, one each for the two data-collection batches comprising 14 502 and 19 496 movies for batches 1 and 2, respectively, thereby assigning the two batches to different exposure groups. In-movie motion was corrected using Patch Motion, saving the images in 16-bit floating point, followed by estimation of micrograph contrast transfer function (CTF) by Patch CTF with default settings. The two micrograph sets were then input into a single Micrograph Junk Detector job with default settings to identify regions of junk within the images. While a high number of micrographs contained features that resembled foil hole-edge (72.5%) and extrinsic ice defects (such as small ice crystals or ethane contaminants; 87.5%), the image area that these took up was relatively low at 4.9% and 3.3%, respectively. Using Manually Curate Exposures, poor-quality micrographs were rejected by using the criteria max CTF fit resolution (Å) = 5, max Intrinsic Ice Defect Area (%) = 10, max Total full-frame motion distance (pixels) = 20 and max Relative Ice Thickness = 1.2. This left 28 448 accepted micrographs (83.7%) that were enhanced using the Micrograph Denoiser job using default settings to enable higher quality particle picking.

#### 2.1.2. Particle picking and cleanup

Particle picking was performed in two stages: initial blob picking to generate 2D class averages, followed by template picking. To speed up the generation of good 2D class averages, a subset of the accepted micrographs was used initially, using the Exposure Sets Tool, setting Split batch size = 5000. Blob picker was run on these 5000 micrographs using particle diameter 150–180 Å and enabling Pick on denoised micrographs. The Micrograph Junk Detector job was used to automatically reject particle picks that were in regions of junk, and Inspect Particle Picks was used in Cluster mode with Target power score = 100. 730 434 particles were extracted with a box of 350 pixels Fourier cropped to 64 and saved in 16-bit floating point. Extracted particles were 2D classified using a maximum resolution of 10 Å, initial uncertainty factor = 1 and 2D zeropad factor = 1. Eight classes resembling a nucleosome were selected using Select 2D Classes (1). To further enhance the quality and diversity of nucleosome views present in the 2D class averages, remaining poor particles were removed before re-running 2D classification; one-class Ab Initio Reconstruction was run using 20 000 of the selected particles from Select 2D and three-class Ab Initio Reconstruction was run using 1000 of the excluded particles from Select 2D. For this second Ab Initio job, the maximum resolution was set at 30 Å, Number of initial iterations = 20 and Number of final iterations = 30. These settings were used to rapidly generate decoy volumes. All four volumes from Ab Initio jobs were input to Heterogeneous Refinement (1), along with the accepted particles from Select 2D, setting Refinement box size (voxels) = 64. Only one of the resulting volumes resembled a nucleosome, so the associated particles were input to 2D Classification with a maximum resolution of 10 Å, initial uncertainty factor = 1 and 2D zeropad factor = 1 and 30 classes. The best 20 classes were selected using Select 2D Classes (2) and these class averages showed a mixture of views: disk, gyres, dyad and some views titled between those (nomenclature of nucleosome views according to Zhou *et al.*, 2019 ▸). Some of the class averages also showed density attached to the nucleosome that might represent ALC1, but classes that did and did not show this density were included. Template Picker was run on all 28 448 accepted micrographs using a Particle diameter of 140 Å and enabling Pick on denoised micrographs. The Micrograph Junk Detector job was used to automatically reject particle picks that were in regions of junk, and Inspect Particle Picks was used in manual mode to set a Local power score range of 57–223 and an NCC score of >0.48. 7 510 094 particles were extracted with a box of 350 pixels Fourier cropped to 64 and saved in 16-bit floating point. Extracted particles were input to 2D Classification with 400 classes, a maximum resolution of 12 Å, initial uncertainty factor = 0.5 and 2D zeropad factor = 1. The large number of classes and low uncertainty factor were used to improve the separation of diverse types of junk. The best 49 classes were selected using Select 2D Classes and input to Heterogeneous Refinement (2) using the previously generated Ab Initio volumes and setting Refinement box size (voxels) = 64.

#### 2.1.3. Consensus refinement

To enable high-resolution 3D refinement, and to re-center images after alignment in 3D, the 1 951 306 particles from the good Heterogeneous Refinement class were re-extracted with a box size of 416 pixels without Fourier cropping and saved in 16-bit floating point. Due to the asymmetric nature of the Widom 601 DNA sequence used to assemble these nucleosomes (Lowary & Widom, 1998 ▸; Ngo *et al.*, 2015 ▸), Non-Uniform (NU) Refinement (1) with the re-extracted particles, and Good Ab Initio volume with *C*2 symmetry and symmetry relaxation by marginalization over poses were run. Minimize over per-particle scale was enabled, and the Dynamic mask start resolution was set to 1 to effectively disable masking. The resulting map achieved a nominal FSC = 0.143 resolution of 2.45 Å and a cFAR score of 0.83, together indicating the likelihood of good orientational sampling of high-quality images, but the map did not show any density that might belong to ALC1. To further improve the map quality, Global CTF Refinement was run including Tilt, Trefoil, Spherical Aberration, Tetrafoil and Anisotropic Magnification. The resulting particles were input to NU Refinement (2) with the same settings as NU Refinement (1) and with the addition of enabling Optimize over per-particle defocus, Optimize per-group CTF params, Fit Spherical Aberration and Fit Tetrafoil. The resulting map achieved a nominal FSC = 0.143 resolution of 2.26 Å and a cFAR of 0.80. A bimodal distribution of the per-particle scale factors was observed, manifesting as a shoulder in the low-scale region. This often indicates poor quality or junk particles, so Subset Particles (https://guide.cryosparc.com/processing-data/all-job-types-in-cryosparc/particle-curation/job-subset-particles-by-statistic) with subset by per-particle scale, subsetting mode Split by manual threshold and a threshold 1 of 0.8 was run to separate particles in the main peak and shoulder. Cluster 1 (the high scale cluster) contained 1 676 608 particles and was used for downstream processing.

#### 2.1.4. Classification of closed ALC1

Previous work on this dataset identified a class of nucleosomes that had a bound ALC1 with a closed ATPase domain clamping on the DNA (Bacic *et al.*, 2021 ▸), but the maps obtained here so far did not show density for ALC1. A generous solvent mask was generated by using Volume Tools with the volume from NU Refinement (2), a low-pass filter of 15 Å, a binarization threshold of 0.025, Dilation radius (pix) = 25 and selecting Type of output volume = mask. To investigate heterogeneity in the sample, 3D Variability Analysis (1) was run using 100 000 particles and the generous solvent mask. 3D Variability Display was then run in simple mode with Downsample to box size = 128 and the resulting volume series was viewed using *ChimeraX*. One of the modes showed the appearance and disappearance of density bound to the side of the nucleosome. The first and last frame volumes from this mode were used to make a difference map in *ChimeraX* by using the volume subtract command, and small blobs of the remaining density were cleaned up by using the volume eraser tool to leave a volume encompassing only the ALC1 region. This volume was uploaded and imported to *CryoSPARC* and used in a Volume Tools job to generate Focus mask (1) by using a low-pass filter of 15 Å, a Threshold of 0.019, a Dilation radius of 4 and a soft padding width of 15 pixels, and selecting Type of output volume = mask. Because these nucleosomes were enzymatically PARylated, resulting in a heterogeneous pattern of PARylation, ALC1 could potentially bind to both sides of the nucleosome. Therefore, classification was performed on both sides separately. Taking advantage of the volume being aligned along the *C*2 symmetry axis of the nucleosome, Volume Alignment Tools was used on the imported ALC1 volume and Focus mask (1) with 3D rotation Euler angles (° or rad) = 0, 0, 180° to produce Focus mask (2). Focus mask (1) along with the generous solvent mask were used in 3D Classification (1) of the Cluster 1 particles into 40 classes, with Initialization mode = PCA, Filter resolution (Å) = 15 and Class similarity = 0.1. Most of the resulting volumes did not show clear density for ALC1, but a single class appeared to represent the closed state of ALC1, and one contained less well ordered density in the same region but with a different shape. Focus mask (2) was not contained within the generous solvent mask, so a very generous solvent mask was created using the same settings except for a Dilation radius of 100 pixels. 3D Classification (2) was performed on the Cluster 1 particles into 80 classes, using the very generous solvent mask and Focus mask (2) with Initialization mode = PCA, Filter resolution (A) = 15 and Class similarity = 0.1. Most of the resulting volumes did not show clear density for ALC1, but a single class appeared to represent the closed state of ALC1. We will not further discuss processing of the closed ALC1 classes, as they represent the same conformation discussed in previous work (Bacic *et al.*, 2021 ▸, 2024 ▸).

#### 2.1.5. Classification of open ALC1

The volume observed in 3D Classification (1) that had less well ordered density in the ALC1 region was examined in *ChimeraX*, the core nucleosome density was removed using the map eraser tool and the map was smoothed using the volume Gaussian command. This volume was uploaded and imported into *CryoSPARC*, and used in a Volume Tools job to generate Focus mask (3) by using a low-pass filter of 15 Å, a Threshold of 0.05, a Dilation radius of 6 and selecting Type of output volume = mask. In a similar strategy as for the closed ALC1 state, Volume Alignment Tools was used on the imported ALC1 volume and Focus mask (3) with 3D rotation Euler angles (° or rad) = 0, 0, 180 ° to produce Focus mask (4). As the particles with closed ALC1 were already identified in 3D Classifications (1) and (2), these particles were excluded from downstream processing by using Particle Sets Tool and inputting Subset Particles Cluster 1 in the Particles A slot, the two particle sets corresponding to the closed ALC1 classes from Classifications (1) and (2) in Particles slot B, and setting Action = intersect. The remaining particles in A minus B (1 581 548) were then used for 3D Classification (3) into 40 classes using the very generous solvent mask, Focus mask (3) with Initialization mode = PCA, Filter resolution (Å) = 5 and Class similarity = 0.1, O-EM batch size (per class) = 300 and O-EM learning rate = 0.9. Classification (4) was performed with the same settings, but using Focus mask (4). In each of 3D Classifications (3) and (4), there was a single class that contained good density in the region of ALC1 comprising 27 909 and 29 529 particles, respectively. These particles were combined and input to NU Refinement (3) using the selected volume from Classification (3) as an input reference and using the same settings as for NU Refinement (2) except for using *C*1 symmetry. The resulting map had a nominal FSC = 0.143 resolution of 2.82 Å and a cFAR of 0.68 (Supplementary Fig. S1). The map showed density for a small region making contact with the nucleosome acidic patch that was of sufficient quality to assign this as part of ALC1 (see Section 2.2), but the rest of the ALC1 density was of poor, featureless quality. The map was examined in *ChimeraX*, the core nucleosome density was removed by using the map eraser tool and the map was smoothed by using the volume Gaussian command. This volume was uploaded and imported to *CryoSPARC* and used in a Volume Tools job to generate Focus mask (5) by using a low-pass filter of 15 Å, a Threshold of 0.05, a Dilation radius of 6 and selecting Type of output volume = mask. To try and compensate for continuous heterogeneity, Local Refinement (1) was performed using Focus mask (3) with an Initial Lowpass resolution of 8 Å, maximum alignment resolution of 5 Å, enabling Re-center rotations each iteration, Re-center shift each iteration and Use pose/shift Gaussian prior during alignment, along with Standard deviation (°) of prior over rotation = 3 and Standard deviation (Å) of prior over shifts = 2. The resulting map had a nominal FSC = 0.143 resolution of 4.76 Å and showed more continuous density outside of the nucleosome, but was still of poor quality. To remove remaining poor particles, or those that struggled to align locally, 3D Classification (5) into ten classes was performed using the Local Refinement input, Focus mask (3) using Initialization mode = PCA, Filter resolution (Å) = 5, Class similarity = 0.1, O-EM batch size (per class) = 300 and O-EM learning rate 0.9. Five of the output classes (totaling 48 806 particles) showed equivalent strong density in the masked region. To further investigate the heterogeneity that was limiting map clarity in the region of interest, a new mask was made to cover the additional region of density. The volume from Local Refinement (1) was examined in *ChimeraX*, the core nucleosome density was removed by using the map eraser tool and the map was smoothed by using the volume Gaussian command. This volume was uploaded and imported to *CryoSPARC* and used in a Volume Tools job to generate Focus mask (6) by using a Threshold of 0.03, a Dilation radius of 20 and selecting Type of output volume = mask. The selected particles from 3D Classification (5) were subjected to NU Refinement (4) with the same settings as NU Refinement (3) followed by 3DVA (Punjani & Fleet, 2021 ▸) with Focus mask (6) using five modes and a filter resolution of 12 Å. 3D Variability Display was then run in simple mode with Filter Resolution = 6 and Downsample to box size = 128, and the resulting volume series 3DVA (2) was viewed using *ChimeraX*. One of the modes showed the motion of a tube of density, apparently inserting into the minor DNA groove. This appeared to represent two discrete states, rather than continuous heterogeneity (Supplementary Fig. S4). Split Volumes was run using the 3DVA series containing the interesting motion, and the volumes for frames 0 and 19 were input to a two-class 3D Classification (6) of the particles from NU Refinement (4) with Initialization mode = input, Filter resolution (Å) = 10, O-EM batch size (per class) = 200 and O-EM learning rate = 0.3. One class containing 15 740 particles displayed a long helical density inserting into the DNA minor groove.

#### 2.1.6. Final refinement, sharpening and validation

The particles from this last class were subjected to Local Refinement (2) using the same settings as for Local Refinement (1). A generous mask was made from the output volume using Volume Tools with a Lowpass Filter of 15 Å and a Dilation radius of 10 pixels, followed by running the FSC validation job. The global resolution at FSC = 0.143 was estimated at 6.60 Å. The same mask and map were used for Local Resolution and Local Filter jobs, which indicated local resolutions at FSC = 0.5 of ∼5–18 Å within the masked region. The same mask along with the locally refined map and particles were also used in an Orientation Diagnostics job. The local refined map displayed a cFAR of 0.42, indicating the possibility of a moderate preferred orientation that was not present in the consensus refinement, and an SCF* of 0.872 (Supplementary Fig. S2). Finally, the locally refined map was sharpened with a *B* factor of −300 Å^2^ using the Sharpen job.

### 2.2. Model building and refinement

An initial atomic model was prepared using *ChimeraX* version 1.10.1 (Pettersen *et al.*, 2021 ▸) by placing fragments into the map manually, or by using the *matchmaker* command between segments of overlapping sequence in the different fragments, followed by rigid-body fitting of each fragment with the *fitmap* command. All ALC1 fragments were taken from the *AlphaFold*2 prediction (AlphaFold-DB accession code AF-Q86WJ1-F1-v3), except for the fragment containing the RLS built by *ModelAngelo* (Jamali *et al.*, 2024 ▸) from the map from NU Refinement (3) (Supplementary Fig. S1) using the full-length sequence information. The following fragments were placed in this order in the map from Local Refinement (2) (Supplementary Fig. S2): the entire nucleosome from PDB entry 8b0a, ALC1 residues Gln599–Glu626 (Regulatory Linker Segment) built by *ModelAngelo* from the map from NU Refinement (3), ALC1 residues Ser581–Glu609 (linker helix) by their overlap with the previous fragment, ALC1 residues Gly627–Tyr874 (macro domain and super-groove recognition helix) by rigid-body fitting, ALC1 residues Ala267–Tyr580 (C-ATPase lobe) by rigid-body fitting, ALC1 residues Ala28–Val266 (N-ATPase lobe) by rigid-body fitting. Overlapping segments were de-duplicated by removing one of the segments to ensure all fragments followed each other in sequence with no duplicated residue numbers or gaps (other than the N- and C-termini that were trimmed because they were not supported by density). All fragments therefore had termini contiguous in sequence, and rigid-body fitting brought them into close spatial proximity, giving us confidence in the rigid-body fitting solutions. The procedure up to this point is shown as a real-time movie (Supplementary Fig. S5). The ALC1 fragments were finally assigned the same chain ID, and all fragments were combined into a single atomic model of the ALC1–nucleosome complex (initial model).

Flexible fitting was performed by interactive molecular dynamics in *ISOLDE* version 1.10.1 (Croll, 2018 ▸), with secondary-structure restraints, base-pair restraints and reference-model restraints for torsion angles (with the initial model as a reference). Attempting to release reference-model restraints led to a substantial degradation of the model geometry, so all restraints were maintained throughout the flexible fitting.

Final refinement was performed with *phenix.real_space_refine* from the *Phenix* suite version 1.21.2-5419 (Afonine *et al.*, 2018 ▸). The refinement strategy was as follows: reference model restraints set to the input model, refine minimization_global and adp.

A morph between the initial model and this final, refined model is shown as a movie (Supplementary Fig. S6).

Validation metrics of the model and model-to-map fit were calculated with *phenix.validation_cryoem* and *phenix.molprobity*. *Q*-scores (Pintilie *et al.*, 2020 ▸) were calculated with the *QScore* extension to *ChimeraX*. Summary statistics of atomic *Q*-scores and *B* factors were calculated with *R* version 4.5.0 (R Core Team, 2025 ▸).

### 2.3. Conservation analysis

The four sequences of ALC1 orthologues with reviewed status available in UniProt at the time of writing (*Homo sapiens*, Q86WJ1; *Mus musculus*, Q9CXF7; *Bos taurus*, Q3B7N1; *Danio rerio*, Q7ZU90) were aligned with *MUSCLE* version 5.2 (Edgar, 2022 ▸). The resulting multiple sequence alignment (MSA) was used to build a profile hidden Markov model (pHMM) using *hmmbuild* from the *HMMER* suite version 3.4 (Eddy, 2011 ▸). This pHMM was used to search for ALC1 orthologues in the reference proteomes of the following model organisms: *Gallus gallus* (UP000000539), *Xenopus laevis* (UP000186698), *Drosophila melanogaster* (UP000000803), *Arabidopsis thaliana* (UP000006548) and *Saccharomyces cerevisiae* (UP000002311). These model organisms were chosen as representatives of vertebrate taxa likely to have an ALC1 orthologue. The proteome of *S. cerevisiae* was used as a negative control in which we do not expect to find an ALC1 orthologue, because this organism does not have any PARP enzymes. The proteome of *A. thaliana* was included as a non-animal but multi-cellular outgroup. The search was performed using *hmmsearch* with a filter to report matches with an *E*-value < 10^−100^. Structure predictions of the top hits from each reference proteome were obtained from AlphaFold-DB when available (Varadi *et al.*, 2022 ▸), or computed with DeepMind’s *AlphaFold*3 server otherwise (Abramson *et al.*, 2024 ▸), and inspected individually. From these structure predictions, plausible ALC1 orthologues were identified based on the following criteria: a general architecture with two ATPase lobes, a linker and a macro domain (in this order from N- to C-terminus), the presence of Arg residues in the nonhelical regions of the linker as putative Arg-anchor residues defining the RLS, and the presence of an α-helix about 40 residues long just upstream of the macro domain as a putative single α-helix (SAH) motif. These criteria retained sequences from *G. gallus* and *A. thaliana*, and excluded the sequence from *X. laevis* that did not exhibit a recognizable putative SAH. Despite having a PARP enzyme, *D. melanogaster* does not seem to have an ALC1 orthologue, as the top hits from the pHMM search against its proteome are the remodelers ISWI and CHD1. The sequences of these two additional plausible ALC1 orthologues were aligned with the initial four sequences to produce a new MSA using *MUSCLE*. This final MSA was used to color the SAH in our structure by sequence conservation using *ChimeraX*. The figure of the final MSA was produced with *Aliview* (Larsson, 2014 ▸). The final MSA is provided as Supplementary Data S1.

### 2.4. Search of chromatin remodeling-associated proteins containing single α-helix motifs

To identify chromatin remodeling-associated proteins containing single α-helix (SAH) motifs, we cross-referenced chromatin remodeling-associated proteins from the UniProt database to the CSAH database (Dudola *et al.*, 2017 ▸). Proteins annotated with the Gene Ontology (GO) term ‘GO:0006338’ (chromatin remodeling) were retrieved from UniProt, yielding 754 369 entries (as of 28 February 2026). This dataset was filtered against the CSAH database (CSAHDB, release 2025-03) to identify proteins with predicted SAH segments. The CSAHDB applies a consensus-based prediction strategy combining the *FT_CHARGE* and *SCAN*4*CSAH* algorithms to detect SAH motifs characterized by regularly alternating clusters of oppositely charged residues (Arg, Lys and Glu), which promote the formation of stable monomeric helices in solution. The intersection of GO-annotated chromatin remodeling-associated proteins with the 1191 CSAHDB entries resulted in 39 unique proteins. This list of chromatin remodeling-associated proteins containing a SAH motif is provided as Supplementary Data S2. Giving priority to the human orthologue when listed, 14 representatives of these proteins were then individually examined by consulting their UniProt annotations and *AlphaFold*2 predictions from AlphaFold-DB to determine whether they harbor a histone PTM reader domain next to the predicted SAH.

## 3. Results

### 3.1. Micrograph and particle curation

The cryo-EM dataset for ALC1 bound to an enzymatically PARylated nucleosome, publicly available as EMPIAR-10739 (Bacic *et al.*, 2021 ▸), was reanalyzed with the initial goal to resolve its heterogeneity and write an educational case study for the *CryoSPARC* Guide (https://guide.cryosparc.com). This new analysis was also motivated by recent new developments in *CryoSPARC* since the original analysis by Bacic *et al.* (2021 ▸) had proven difficult in large part due to heterogeneity and to less visual feedback provided by the image-processing tools available at the time. Notably, only a stringent particle-picking strategy using the micrograph denoiser from *Topaz* (Bepler *et al.*, 2020 ▸) to aid manual picking of a training set, followed by training and picking with *Topaz* (Bepler *et al.*, 2019 ▸), had yielded reconstructions with density recognizable as ALC1.

During this reanalysis, careful curation of the micrographs was performed with two new tools in *CryoSPARC*: the Micrograph Denoiser (https://guide.cryosparc.com/processing-data/all-job-types-in-cryosparc/exposure-curation/job-micrograph-denoiser-beta) and the Micrograph Junk Detector (https://guide.cryosparc.com/processing-data/all-job-types-in-cryosparc/exposure-curation/job-micrograph-junk-detector-beta). A two-pass particle-picking strategy was applied on the denoised micrographs: blob picking; particle curation by 2D classification, *ab initio* reconstruction and heterogeneous refinement; and template picking. The selected 2D classes included recognizable nucleosomes with high-resolution features, irrespective of whether or not they showed signal attributable to ALC1 outside of the nucleosome. This was to avoid the risk of introducing an orientation bias in viewing directions where the extra signal produces more contrast. Particle curation resulted in a set of particles that gave a high-resolution nucleosome reconstruction that did not appear to have density recognizable as ALC1. The lack of ALC1 density at this stage could be explained by a combination of the nucleosome itself dominating particle image alignment, substoichiometric ALC1 binding and/or a distribution of ALC1 bound at different locations and/or with different conformations that were averaged out in the reconstruction. These procedures are described in detail in Sections 2.1.1 and 2.1.2 and summarized in Fig. 3 ▸.

### 3.2. Replication of the closed ALC1–nucleosome complex reconstruction

The conformation of ALC1 with its ATPase domain closed and clamping on the nucleosomal DNA was first reported in Bacic *et al.* (2021 ▸) and was found following stringent particle picking and extensive 3D classification. Classification of this complex was also performed as part of reprocessing this EMPIAR-10739 dataset as a case study for the *CryoSPARC* Guide (https://guide.cryosparc.com/processing-data/tutorials-and-case-studies/case-study-end-to-end-and-exploratory-processing-of-a-motor-bound-nucleosome-empiar-10739). This procedure involved 3D variability analysis (3DVA) on the particles from the consensus refinement and masked 3D classifications, as detailed in Section 2.1.4. The same complex as described in Bacic *et al.* (2021 ▸) (PDB entry 7otq; EMDB entry EMD-13065) was also obtained in this reanalysis (Fig. 3 ▸; Classify closed ALC1, yellow background). We were even able to significantly improve this reconstruction, which is described in detail in the case study in the *CryoSPARC* Guide. Although successful replication of this result is satisfying, the improved reconstruction does not bring new biological information in terms of global conformation or visible domains (the macro domain and linker are still not resolved) compared with the later 3.0 Å resolution structure of ALC1 bound to a site-specifically PARylated nucleosome (Bacic *et al.*, 2024 ▸; PDB entry 8b0a; EMDB entry EMD-15777). Therefore, we will not further describe this complex with a closed ATPase domain here.

### 3.3. Extensive 3D classification identifies a complex with ALC1 in an open conformation

The original analysis of this dataset by Bacic *et al.* (2021 ▸) followed three strategies. First, via multiple rounds of hierarchical 3D classification in *RELION*, the alignments degraded as the set of particles was iteratively split into more classes, each one populated by fewer particles. Perhaps not surprisingly, this strategy did not produce any interpretable reconstruction. The second strategy used 3DVA in cluster mode (*CryoSPARC* version 3.2) and isolated a set of particles corresponding to the closed ATPase domain state. The third strategy used *CryoDRGN*, recently published at the time, and produced reconstructions of multiple conformational states of ALC1 (map series deposited as EMD-13070), all consistent with expectations based on prior knowledge: (i) density recognizable as the ATPase domain was located similarly as in structures of other remodeler–nucleosome complexes and (ii) density recognizable as the macro domain was located near the site where the H3 tail protrudes from the nucleosome, consistent with the facts that the macro domain binds to PAR chains and that the main site of ADP-ribosylation in nucleosomes is Ser10 in H3 (Bonfiglio *et al.*, 2017 ▸). However, none of the maps from *CryoDRGN* had sufficient resolution to rigid-body fit the domains of ALC1 confidently, and the distribution of latent space coordinates of the particles did not reveal discrete clusters that could be separately subjected to conventional refinement and reconstruction.

In this reanalysis, a first reconstruction was obtained that showed clear density at the RLS of ALC1 interacting with the acidic patch [Fig. 4 ▸, Classify open ALC1, Non-Uniform Refinement (3), red background; Supplementary Fig. S1]. This density supported assignment of the sequence of the RLS using automated model building by *ModelAngelo* (Jamali *et al.*, 2024 ▸). Another reconstruction was eventually obtained, of lower resolution (6.6 Å over the region assigned as ALC1), but in which all domains of ALC1 could be identified [Fig. 4 ▸, Final map, Local Refinement (2), pink background; Supplementary Fig. S2]. This required a combination of 3DVA and deep 3D classification (with no alignment) using wide masks encompassing the space occupied by ALC1. The procedure is detailed in Section 2.1.5 and summarized in Figs. 3 ▸ and 4 ▸. It was critical to consider the fact that ALC1 could bind on either side of the nucleosome due to the heterogeneous PARylation pattern on the nucleosome. Therefore, 3D classification needed to be performed for both sides separately. Note that this was not performed as a single classification with *C*2 symmetry expansion because we wanted to first ensure that the asymmetric DNA sequence had not visibly influenced the binding of ALC1, and that its conformation was the same on both sides. The resulting reconstructions were realigned to match ALC1 on a single side. Although this will have contributed to blurring the asymmetric DNA sequence, it improved the quality of the density for ALC1.

### 3.4. The open conformation of ALC1 may be an intermediate between the auto-inhibited and active states

**Figure 5:**
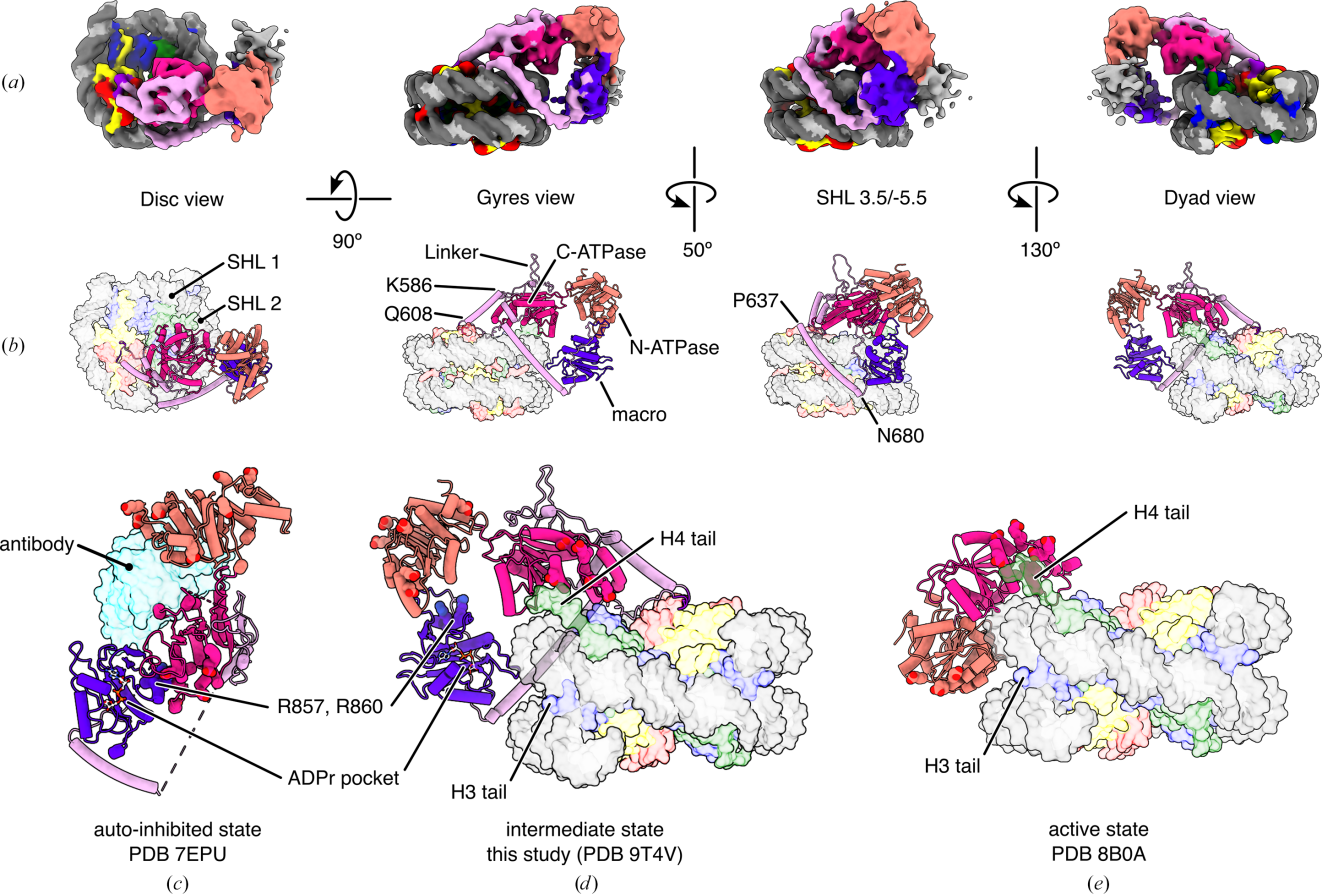
Structure of ALC1 in an open conformation, bound to a PARylated nucleosome. (*a*) Cryo-EM map colored by chain assignment, following the color codes from Fig. 1 ▸(*a*) for the nucleosome and Fig. 2 ▸(*a*) for ALC1. (*b*) Atomic model, shown in the same orientations and with the same color code as the map shown in (*a*). (*c*) Structure of the auto-inhibited state. The antibody used for crystallization is shown as a translucent cyan surface. From PDB entry 7epu. (*d*) Structure of the intermediate state (dyad view). From this study. (*e*) Structure of the active state. From PDB entry 8b0a. In (*c*), (*d*) and (*e*), the ATPase residues mutated in the study by Lehmann *et al.* (2017 ▸) are shown as spheres. In (*c*) and (*d*), the macro domain residues Arg857 and Arg860, found mutated in cancer, are shown as spheres and labeled, and the ADPr-binding pocket of the macro domain is indicated by an ADPr molecule (shown in ball-and-stick representation) modeled by structural superimposition of the structure of the Af1521–ADPr complex from PDB entry 2bfq (the Af1521 protein is not shown for clarity). In (*b*), (*d*) and (*e*), the nucleosome is shown as a translucent surface for clarity. See also Supplementary Fig. S2 for the validation of this cryo-EM map (including local resolution and orientation distribution). See also Supplementary Fig. S3 for a movie of an interpolation between the closed and open conformations of nucleosome-bound ALC1.

This extensive curation and classification procedure identified an ALC1–nucleosome complex with the ATPase domain of ALC1 in an open conformation and in which all domains of ALC1 are finally visualized in the context of a nucleosome complex (Fig. 5 ▸*a*). The final classified particle set contains 15 740 particles, which constitutes only ∼1% of the curated particles in the consensus refinement. This reconstruction has a overall resolution of 6.6 Å over the region assigned as ALC1, and the map quality for the N-ATPase lobe and macro domain is relatively poor compared with the nucleosome and C-ATPase lobe, as reflected by local resolution estimates ranging from 5 to 15 Å (Supplementary Fig. S2). This likely indicates unresolved heterogeneity, but attempts to further subclassify did not improve the map quality in these regions, probably limited by the small number of particles remaining. This set of particles also presents under-sampling of some orientations (Supplementary Fig. S2), although the map does not show signs of severe anisotropy. The map still enabled unambiguous rigid-body fitting of all domains (for details, see Section 2.2 and Supplementary Fig. S5), allowing interpretation of their relative orientations and interactions at the secondary-structure level.

This structure presents unique features not observed in the structures of the auto-inhibited and active states (Figs. 5 ▸*b*–5 ▸*e*). Most strikingly, the two ATPase lobes are found in an open conformation and do not clamp the DNA (Fig. 5 ▸*d*). The N-ATPase lobe does not interact with the DNA and instead interacts with the macro domain (compare Figs. 5 ▸*d* and 5 ▸*e*), consistent with previous evidence of this interaction from cross-linking mass spectrometry (Lehmann *et al.*, 2017 ▸). The C-ATPase lobe interacts with the H4 tail and with the DNA, but closer to SHL 3 (Fig. 5 ▸*b*), a location clearly distinct from SHL 2 where this domain lies in the active state. This is consistent with our previous analysis of this cryo-EM dataset with *CryoDRGN*, which had revealed that ALC1 samples the nucleosome at a range of locations between SHL 1 and SHL 3 (Bacic *et al.*, 2021 ▸). The RLS interacts with the acidic patch and is thus detected in a non-cross-linked structure for the first time. The macro domain interacts with both the N-ATPase lobe and the DNA. The macro domain most likely also interacts with the PAR chains that are too heterogeneous and flexible to be visualized. This is supported by the observations that the ADP ribose (ADPr)-binding pocket of the macro domain is exposed in the auto-inhibited state (Fig. 5 ▸*c*) and points towards the tail of H3 (where H3-Ser10, the main site of histone PARylation, is located) in this new structure (Fig. 5 ▸*d*). Because the ATPase domain is not clamped on the DNA at SHL 2, this structure does not represent a catalytically active state. The conformation of ALC1 observed here is however also markedly different from its auto-inhibited state: the macro domain interacts with the N-ATPase lobe instead of the C-ATPase lobe (compare Figs. 5 ▸*c* and 5 ▸*d*). This is consistent with previous evidence from small-angle X-ray scattering and cross-linking mass spectrometry showing that the macro domain could interact with both ATPase lobes (Lehmann *et al.*, 2017 ▸). The sample contained ADP–BeF_3_, an ATP analog that traps remodelers on nucleosomes, presumably in the closed ATPase domain conformation (Ren *et al.*, 2019 ▸). At a 333-fold molar excess over ALC1 (1 m*M* ADP–BeF_3_, 3 µ*M* ALC1) and with a long incubation time of 50 min (detailed in Bacic *et al.*, 2021 ▸), we expected these conditions to yield mostly the closed conformation of ALC1. This new structure might therefore represent an intermediate state of ALC1, possibly during the release of its auto-inhibition or during the ADP/ATP-exchange step that requires an open ATPase domain (since the nucleotide-binding site is at the interface of the two lobes). This structure alone is however not sufficient to determine whether this state is ‘on- or off-pathway’, which will require experimental investigation by functional assays.

### 3.5. The linker of ALC1 contains a single α-helix motif

This new structure reveals that the linker of ALC1 is in fact much less disordered than previously thought. Residues Ala507–Lys519 and Leu522–Ala527 form two short α-helices that rest on the surface of the C-ATPase lobe. Residues Lys586–Gln608 form an α-helix leading into the RLS, likely providing enough rigidity in the linker to restrict the conformational freedom of the RLS (residues Gly610–Gly617) and help point it at the acidic patch. Residues Ser528–Ser585 do not form regular secondary structures, but seem to interact with the C-ATPase lobe. This segment of the linker might provide some flexibility to balance the rigid helix Lys586–Gln608, possibly facilitating the interaction between the RLS and the acidic patch by helping to bridge a variable distance between the acidic patch and the C-ATPase lobe before its interactions with the H4 tail and DNA restrict its movement.

**Figure 6:**
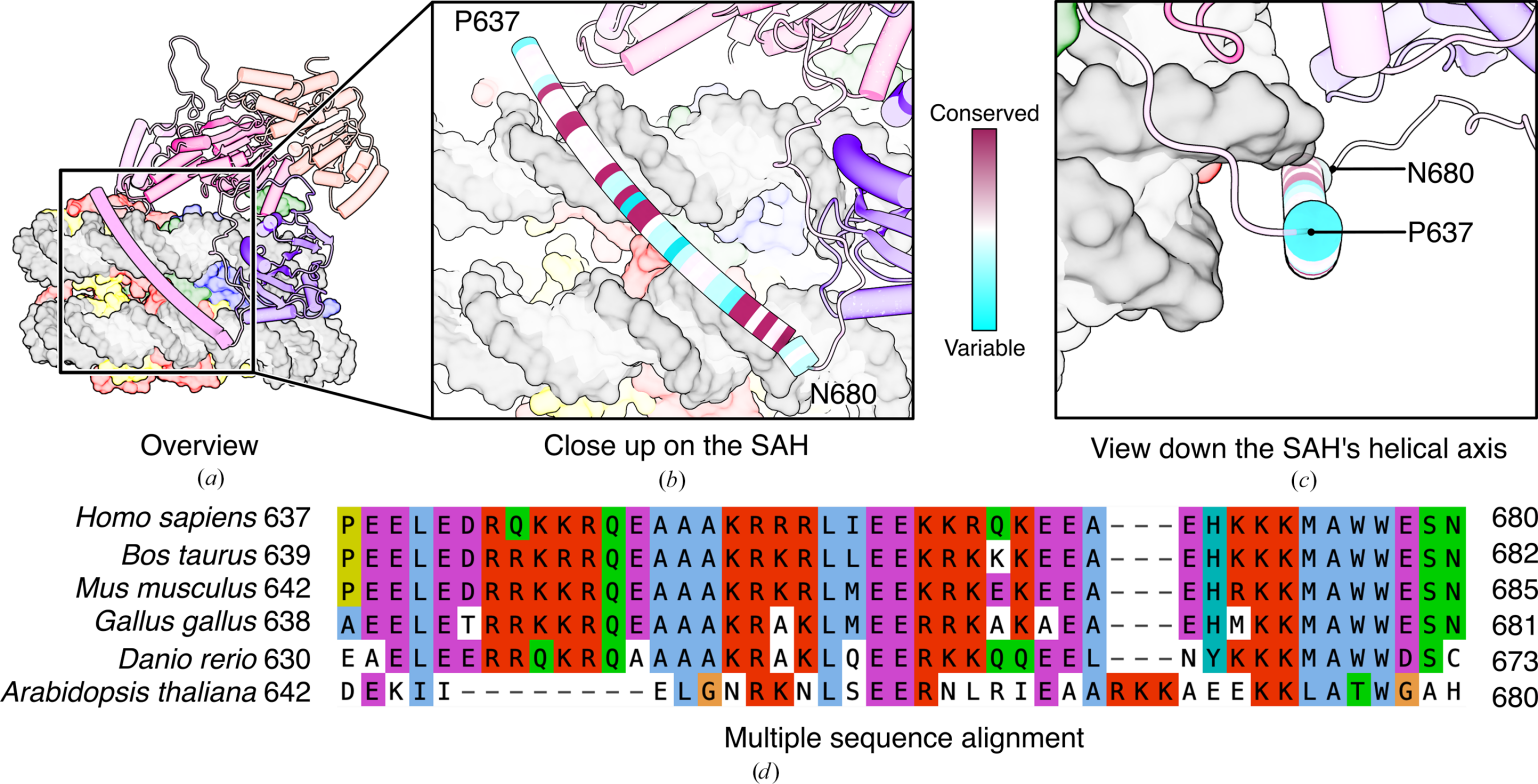
The single α-helix (SAH) motif of ALC1. (*a*) Overview of the SAH of ALC1 in the context of the entire structure. The color code is the same as in Fig. 5 ▸. The rest of ALC1 and the nucleosome are slightly translucent for clarity. (*b*, *c*) Close-up views of the SAH (*b*) seen from the side and (*c*) looking down the helical axis. The SAH is colored by conservation. (*d*) Multiple sequence alignment of ALC1 orthologues verified by structure predictions to have a similar domain architecture to human ALC1, including a putative SAH motif just upstream of the macro domain.

Besides the open conformation of the ATPase domain described above, the next most striking feature of this structure is a long α-helix from residues Pro637 to Asn680 of ALC1. This helix was predicted by *AlphaFold*2 (Fig. 2 ▸*b*) and observed in the crystal structure of the auto-inhibited state (Fig. 2 ▸*c*). Closer inspection of this helix indicates that it has the sequence signature of the ‘single α-helix’ (SAH) motif: repeats of four acidic residues followed by four basic residues (Süveges *et al.*, 2009 ▸; Wolny *et al.*, 2017 ▸; Dudola *et al.*, 2017 ▸). The SAH of ALC1 connects its RLS and its macro domain, and the locations of these two structural elements in the new structure make the SAH reach across both DNA gyres (Figs. 6 ▸*a* and 6 ▸*b*). This helix is conserved among ALC1 orthologues from vertebrates (Figs. 6 ▸*b*–6 ▸*d*). It is rich in basic residues, with six arginines and nine lysines together representing 34% of its 44 residues. These basic residues are distributed along the entire length of the helix (Fig. 6 ▸*d*), and their occurrence as 3–4 consecutive residues means that they point in all directions around the helical axis, since an α-helix has 3.6 residues per helical turn. These properties may promote electrostatic interactions with the nearby DNA, which could in turn explain the apparent alignment of the SAH with the DNA grooves.

In our previous study, we observed that a macro domain construct starting at residue Leu613, therefore encompassing the entire SAH, shifted unmodified nucleosomes in electrophoretic mobility shift assays (see Fig. 1*a* in Bacic *et al.*, 2021 ▸). This suggests that the SAH might in fact interact with nucleosomal DNA.

### 3.6. Other chromatin-remodeling factors contain an SAH motif

To evaluate whether the SAH motif of ALC1 could have a functional role beyond simply connecting domains, we searched for SAH motifs in proteins linked to chromatin remodeling. In brief, this search consisted in finding the intersection between the CSAH database (Dudola *et al.*, 2017 ▸) and UniProt entries annotated with the Gene Ontology ‘chromatin remodeling’ (detailed in Section 2.4). This identified 39 proteins (Supplementary Data S2). In addition to ALC1/CHD1L, the list includes other remodelers such as yeast Chd1 and the SWC3 subunit of the SWR1 remodeling complex; the A subunit of the CAF-1 complex involved in chromatin replication; histone PTM writers such as the E3 ubiquitin ligase RNF168, histone methyltransferases SET1, SET9 and SET26; and histone PTM readers such as the bromodomain-containing protein BAZ1A.

Finding predicted SAH motifs in proteins associated with chromatin remodeling and histone PTMs suggests that this motif could have functional roles in combination with other domains. Closer examination indicates that none of these 39 proteins harbor a histone PTM reader domain immediately next to its predicted SAH motif. However, SET-9 and SET-26 from *Caenorhabditis elegans* have their SET domain next to their predicted SAH, and human RNF168 has its zinc-finger domain close to its SAH.

## 4. Discussion

### 4.1. On finding the unexpected

By revisiting the publicly available cryo-EM dataset from our previous study, we discovered an ALC1–nucleosome complex in which the ATPase domain is in an open conformation, possibly representing an intermediate between the auto-inhibited and active states of the remodeler. Functional assays are still necessary to assess whether this state is part of the activation process or remodeling cycle, and this new model comprising all domains will help formulate testable hypotheses. Based on our experience working on ALC1, we believe that no biochemical manipulation would have enabled us to trap and enrich this conformation. Therefore, biochemical reconstitution mimicking an *in vivo* situation (however complex) as closely as possible, paired with extensive classification during image processing, is a strategy that could be more commonly employed when working on chromatin complexes or other heterogeneous systems.

### 4.2. Possible function of the SAH motif of ALC1

A discovery from this newly visualized state of ALC1 is that the segment of its linker from residues Pro637 to Asn680, connecting the RLS to the macro domain, is in fact a single α-helix (SAH) motif.

The SAH could play an integral part in the activation mechanism, for example by acting as a mechanical lever to help dissociate the macro domain from the ATPase domain. This hypothesis is supported by the fact that residues Leu640–Glu667 of the linker are already folded as a helix in the auto-inhibited state, while residues Ala668–Asn680 are not (Fig. 2 ▸*c*). This Ala668–Asn680 segment is likely to fold upon interaction with nucleosomal DNA, completing the SAH. This transition from coil to helix would stiffen this last segment, which might help pull the macro domain off the ATPase domain. Such a mechanical role seems plausible given that an SAH motif was found in myosins, a distinct group of ATP-dependent motor proteins, in which it is part of the ‘lever arm’ and acts as a ‘constant-force spring’ contributing to force generation during the ATP-hydrolysis cycle (Knight *et al.*, 2005 ▸; Wolny *et al.*, 2014 ▸; Barnes *et al.*, 2019 ▸). In further support of a functional role of the SAH, the COSMIC database (Sondka *et al.*, 2024 ▸) lists several mutations associated with cancers in this residue range: eight missense mutations (P637L, R647K, E649Q, L657F, I658V, K661E, A668T, M674T) and one single-residue deletion (Arg656).

The following sequence of events for the activation of ALC1 is compatible with all functional and structural evidence available: (i) binding of the macro domain to PAR chains recruits ALC1 to a nucleosome; (ii) the H4 tail out-competes the macro domain for binding to the C-ATPase lobe, the SAH positions the RLS so that it interacts with the acidic patch and these interactions pull the C-ATPase lobe and macro domain apart, forcing them to dissociate; (iii) the C-ATPase lobe finds its correct orientation because it gets anchored by three points: its interactions with the H4 tail and the DNA, and the interaction of the RLS with the acidic patch; (iv) the macro domain ‘rolls over’ to interact with the N-ATPase lobe instead; (v) maintained by its interaction with PAR chains and by the stiff SAH, the macro domain eventually dissociates from the N-ATPase lobe, allowing it to close the ATPase domain’s clamp on the DNA at SHL 2 (a hallmark of the active state). Of note, CHD1, the closest paralogue of ALC1, undergoes similarly large conformational transitions involving interactions between several of its domains and regulatory structural elements (James & Farnung, 2025 ▸). These hypotheses on the activation mechanism of ALC1 will require experimental testing in future functional studies.

### 4.3. SAH motifs in other chromatin-binding factors

While SAH motifs have been predicted and experimentally characterized in other biological contexts (for example, myosins), experimental evidence seems to be largely lacking for SAHs in proteins involved in chromatin remodeling. These regions might remain under-explored because of their low structural complexity and because their functions may not require tight spatial confinement, which hinders high-resolution structural determination. It is also possible that as with ALC1, some SAH motifs are fully folded only in the context of a complex, making their experimental identification more difficult.

The example of ALC1, with its SAH located just upstream of its macro domain, suggests a more general class of regulatory structural elements in chromatin-binding factors: an SAH motif directly connected to any histone PTM reader domain. Such arrangements of structural elements may be searched systematically by bioinformatic means. Our initial attempt uncovered 39 chromatin remodeling-associated proteins containing a predicted SAH motif (Supplementary Data S2); however, none of them harbor a histone PTM reader domain immediately upstream or downstream of the predicted SAH motif. Broader searches are warranted, for example among histone PTM writers and readers, or among DNA repair factors.

### 4.4. Possible role of the nucleosome super-groove

One notable structural feature of the nucleosome is that the major and minor grooves of the DNA perfectly align across the two gyres, forming what has been termed a super-groove spanning both gyres. It was demonstrated that dimeric synthetic DNA minor-groove binders can specifically recognize DNA sequences exposed in the super-groove (Edayathumangalam *et al.*, 2004 ▸). Some dimeric pioneer transcription factors are suspected to target the super-groove, although there has been no direct evidence to date (Zhu *et al.*, 2018 ▸; Makowski *et al.*, 2020 ▸). Haspin, a histone H3 kinase involved in mitosis, has been demonstrated to bind to the cavity between the two gyres at the major super-groove at SHL 5.5/−2.5 (Hicks *et al.*, 2025 ▸). With this single example of recognition of the super-groove by a naturally occurring protein described at the structural level to date, the prevalence of this mode of recognition among chromatin-binding proteins remains unknown.

We find it intriguing that the SAH motif of ALC1 runs parallel to a super-groove (Fig. 6 ▸) and seems to reposition to the next super-groove upon conformational change of the rest of the protein (Supplementary Fig. S4) in a manner that does not look random. Basic residues in the SAH may promote its interaction with nucleosomal DNA throughout the large conformational change undergone by ALC1 during its activation, although the low resolution of this new reconstruction cannot ascertain this.

It seems unlikely that the SAH would be necessary for recognition of the nucleosome, since other structural elements of ALC1 interact with multiple nucleosomal epitopes: the macro domain with PAR chains, the RLS with the acidic patch, and the ATPase domain with the H4 tail and the DNA at SHL 2. Simultaneously probing the H4 tail and the acidic patch guarantees that the bound substrate is a nucleosome, and the directionality of nucleosome sliding is established by the PARylation pattern (Bacic *et al.*, 2024 ▸). However, under the hypothesis stated above that the SAH could act as a mechanical lever to pull the macro domain off of the ATPase domain, precise positioning of the SAH by a super-groove could play a role in the activation process. Addressing these questions will also require further experimental investigation.

### 4.5. On the importance of making raw data publicly available

We note and emphasize that the findings presented here would most likely not have been made had we not deposited the dataset from our 2021 study in EMPIAR (Iudin *et al.*, 2023 ▸). We hope this report will encourage other structural biologists to deposit the raw data supporting their structural models into public databases, and that it will also encourage downstream users of public datasets to reach out to the depositors when new discoveries are made. Fruitful collaborations can ensue, as we hope we have demonstrated here.

## Supplementary Material

PDB reference: ALC1–nucleosome complex in an activation intermediate state, 9t4v

EMDB reference: ALC1–nucleosome complex in an activation intermediate state, EMD-55533

EMDB reference: reconstruction used to model the regulatory linker segment with *ModelAngelo*, EMD-55534

Suppementary Figures and Table. DOI: 10.1107/S2059798326004158/cb5156sup1.pdf

Supplementary Figure S3. Movie of an interpolation between the closed and open conformations of nucleosome-bound ALC1. DOI: 10.1107/S2059798326004158/cb5156sup2.mp4

Supplementary Figure S4. Movie of 3DVA (2). DOI: 10.1107/S2059798326004158/cb5156sup3.mp4

Supplementary Figure S5. Movie of the rigid-body fitting procedure. DOI: 10.1107/S2059798326004158/cb5156sup4.mp4

Supplementary Figure S6. Movie of the flexible fitting procedure. DOI: 10.1107/S2059798326004158/cb5156sup5.mp4

Supplementary Data S1. Final multiple sequence alignment. DOI: 10.1107/S2059798326004158/cb5156sup6.zip

Supplementary Data S2. List of chromatin remodeling associated proteins predicted to contain an SAH motif. DOI: 10.1107/S2059798326004158/cb5156sup7.zip

Supplementary Data S3. ChimeraX command file to set up a session with the same color code and style as in the figures. DOI: 10.1107/S2059798326004158/cb5156sup8.txt

## Data Availability

The cryo-EM dataset re-analyzed in this study is publicly available as EMPIAR-10739. The reconstruction of the ALC1–nucleosome complex in an activation intermediate state is deposited in the EMDB with accession code EMD-55533 [the map series from 3DVA (2) is deposited in the same entry as additional maps] and the corresponding atomic model is deposited in the PDB with accession code pdb_00009t4v. The reconstruction used to model the RLS with *ModelAngelo* is deposited in the EMDB with accession code EMD-55534.

## References

[bb1] Abramson, J., Adler, J., Dunger, J., Evans, R., Green, T., Pritzel, A., Ronneberger, O., Willmore, L., Ballard, A. J., Bambrick, J., Bodenstein, S. W., Evans, D. A., Hung, C.-C., O’Neill, M., Reiman, D., Tunyasuvunakool, K., Wu, Z., Žemgulytė, A., Arvaniti, E., Beattie, C., Bertolli, O., Bridgland, A., Cherepanov, A., Congreve, M., Cowen-Rivers, A. I., Cowie, A., Figurnov, M., Fuchs, F. B., Gladman, H., Jain, R., Khan, Y. A., Low, C. M. R., Perlin, K., Potapenko, A., Savy, P., Singh, S., Stecula, A., Thillaisundaram, A., Tong, C., Yakneen, S., Zhong, E. D., Zielinski, M., Žídek, A., Bapst, V., Kohli, P., Jaderberg, M., Hassabis, D. & Jumper, J. M. (2024). *Nature*, **630**, 493–500.

[bb2] Afonine, P. V., Poon, B. K., Read, R. J., Sobolev, O. V., Terwilliger, T. C., Urzhumtsev, A. & Adams, P. D. (2018). *Acta Cryst.* D**74**, 531–544. 10.1107/S2059798318006551 PMC6096492 29872004

[bb3] Ahel, D., Hořejší, Z., Wiechens, N., Polo, S. E., Garcia-Wilson, E., Ahel, I., Flynn, H., Skehel, M., West, S. C., Jackson, S. P., Owen-Hughes, T. & Boulton, S. J. (2009). *Science*, **325**, 1240–1243. 10.1126/science.1177321 PMC3443743 19661379

[bb4] Armache, J.-P., Gamarra, N., Johnson, S. L., Leonard, J. D., Wu, S., Narlikar, G. J. & Cheng, Y. (2019). *eLife*, **8**, e46057. 10.7554/eLife.46057 PMC6611695 31210637

[bb5] Ayala, R., Willhoft, O., Aramayo, R. J., Wilkinson, M., McCormack, E. A., Ocloo, L., Wigley, D. B. & Zhang, X. (2018). *Nature*, **556**, 391–395. 10.1038/s41586-018-0021-6 PMC5937682 29643506

[bb6] Bacic, L., Gaullier, G., Mohapatra, J., Mao, G., Brackmann, K., Panfilov, M., Liszczak, G., Sabantsev, A. & Deindl, S. (2024). *Nat. Commun.* **15**, 1000. 10.1038/s41467-024-45237-8 PMC10837151 38307862

[bb7] Bacic, L., Gaullier, G., Sabantsev, A., Lehmann, L. C., Brackmann, K., Dimakou, D., Halic, M., Hewitt, G., Boulton, S. J. & Deindl, S. (2021). *eLife*, **10**, e71420. 10.7554/eLife.71420 PMC8463071 34486521

[bb8] Baker, R. W., Reimer, J. M., Carman, P. J., Turegun, B., Arakawa, T., Dominguez, R. & Leschziner, A. E. (2021). *Nat. Struct. Mol. Biol.* **28**, 71–80. 10.1038/s41594-020-00528-8 PMC7855068 33288924

[bb9] Barnes, C. A., Shen, Y., Ying, J., Takagi, Y., Torchia, D. A., Sellers, J. R. & Bax, A. (2019). *J. Am. Chem. Soc.* **141**, 9004–9017. 10.1021/jacs.9b03116 PMC6556874 31117653

[bb10] Bepler, T., Kelley, K., Noble, A. J. & Berger, B. (2020). *Nat. Commun.* **11**, 5208. 10.1038/s41467-020-18952-1 PMC7567117 33060581

[bb11] Bepler, T., Morin, A., Rapp, M., Brasch, J., Shapiro, L., Noble, A. J. & Berger, B. (2019). *Nat. Methods*, **16**, 1153–1160. 10.1038/s41592-019-0575-8 PMC6858545 31591578

[bb12] Bilokapic, S., Suskiewicz, M. J., Ahel, I. & Halic, M. (2020). *Nature*, **585**, 609–613. 10.1038/s41586-020-2725-7 PMC7529888 32939087

[bb13] Bonfiglio, J. J., Fontana, P., Zhang, Q., Colby, T., Gibbs-Seymour, I., Atanassov, I., Bartlett, E., Zaja, R., Ahel, I. & Matic, I. (2017). *Mol. Cell*, **65**, 932–940. 10.1016/j.molcel.2017.01.003 PMC5344681 28190768

[bb14] Bowman, G. D. & Deindl, S. (2019). *Science*, **366**, 35–36. 10.1126/science.aay4317 31604293

[bb15] Chittori, S., Hong, J., Bai, Y. & Subramaniam, S. (2019). *Nucleic Acids Res.* **47**, 9400–9409. 10.1093/nar/gkz670 PMC6755096 31402386

[bb16] Clapier, C. R. & Cairns, B. R. (2009). *Annu. Rev. Biochem.* **78**, 273–304. 10.1146/annurev.biochem.77.062706.153223 19355820

[bb17] Clapier, C. R., Iwasa, J., Cairns, B. R. & Peterson, C. L. (2017). *Nat. Rev. Mol. Cell Biol.* **18**, 407–422. 10.1038/nrm.2017.26 PMC8127953 28512350

[bb18] Croll, T. I. (2018). *Acta Cryst.* D**74**, 519–530. 10.1107/S2059798318002425 PMC6096486 29872003

[bb19] Dudola, D., Tóth, G., Nyitray, L. & Gáspári, Z. (2017). *Methods Mol. Biol.* **1484**, 25–34. 10.1007/978-1-4939-6406-2_3 27787817

[bb20] Edayathumangalam, R. S., Weyermann, P., Gottesfeld, J. M., Dervan, P. B. & Luger, K. (2004). *Proc. Natl Acad. Sci. USA*, **101**, 6864–6869. 10.1073/pnas.0401743101 PMC406433 15100411

[bb21] Eddy, S. R. (2011). *PLoS Comput. Biol.* **7**, e1002195. 10.1371/journal.pcbi.1002195 PMC3197634 22039361

[bb22] Edgar, R. C. (2022). *Nat. Commun.* **13**, 6968. 10.1038/s41467-022-34630-w PMC9664440 36379955

[bb23] Eustermann, S., Schall, K., Kostrewa, D., Lakomek, K., Strauss, M., Moldt, M. & Hopfner, K.-P. (2018). *Nature*, **556**, 386–390. 10.1038/s41586-018-0029-y PMC6071913 29643509

[bb24] Farnung, L., Ochmann, M. & Cramer, P. (2020). *eLife*, **9**, e56178. 10.7554/eLife.56178 PMC7338049 32543371

[bb25] Farnung, L., Vos, S. M., Wigge, C. & Cramer, P. (2017). *Nature*, **550**, 539–542. 10.1038/nature24046 PMC5697743 29019976

[bb26] Gaullier, G., Roberts, G., Muthurajan, U. M., Bowerman, S., Rudolph, J., Mahadevan, J., Jha, A., Rae, P. S. & Luger, K. (2020). *PLoS One*, **15**, e0240932. 10.1371/journal.pone.0240932 PMC7608914 33141820

[bb27] Gibbs-Seymour, I., Fontana, P., Rack, J. G. M. & Ahel, I. (2016). *Mol. Cell*, **62**, 432–442. 10.1016/j.molcel.2016.03.008 PMC4858568 27067600

[bb28] Gottschalk, A. J., Timinszky, G., Kong, S. E., Jin, J., Cai, Y., Swanson, S. K., Washburn, M. P., Florens, L., Ladurner, A. G., Conaway, J. W. & Conaway, R. C. (2009). *Proc. Natl Acad. Sci. USA*, **106**, 13770–13774. 10.1073/pnas.0906920106 PMC2722505 19666485

[bb29] Gottschalk, A. J., Trivedi, R. D., Conaway, J. W. & Conaway, R. C. (2012). *J. Biol. Chem.* **287**, 43527–43532. 10.1074/jbc.M112.401141 PMC3527939 23132853

[bb30] Han, Y., Reyes, A. A., Malik, S. & He, Y. (2020). *Nature*, **579**, 452–455. 10.1038/s41586-020-2087-1 PMC7319049 32188938

[bb31] He, S., Wu, Z., Tian, Y., Yu, Z., Yu, J., Wang, X., Li, J., Liu, B. & Xu, Y. (2020). *Science*, **367**, 875–881. 10.1126/science.aaz9761 32001526

[bb32] He, Z., Chen, K., Ye, Y. & Chen, Z. (2021). *Cell. Discov.* **7**, 28. 10.1038/s41421-021-00262-5 PMC8079448 33907182

[bb33] Hicks, C. W., Gliech, C. R., Rahman, S., Zhang, X., Eneim, A. S., Vasquez, S. J., Holland, A. J. & Wolberger, C. (2025). *Nat. Struct. Mol. Biol.* **32**, 1030–1037. 10.1038/s41594-025-01502-y PMC12170164 39979508

[bb34] Hu, P., Sun, J., Sun, H., Chen, K., Sia, Y., Xia, X., Xi, Q. & Chen, Z. (2025). *Nature*, **644**, 818–826. 10.1038/s41586-025-09100-0 40468067

[bb35] Iudin, A., Korir, P. K., Somasundharam, S., Weyand, S., Cattavitello, C., Fonseca, N., Salih, O., Kleywegt, G. J. & Patwardhan, A. (2023). *Nucleic Acids Res.* **51**, D1503–D1511. 10.1093/nar/gkac1062 PMC9825465 36440762

[bb36] Jalal, A. S. B., Girvan, P., Chua, E. Y. D., Liu, L., Wang, S., McCormack, E. A., Skehan, M. T., Knight, C. L., Rueda, D. S. & Wigley, D. B. (2024). *Mol. Cell*, **84**, 3871–3884.e9. 10.1016/j.molcel.2024.08.015 PMC7619409 39226902

[bb37] Jamali, K., Käll, L., Zhang, R., Brown, A., Kimanius, D. & Scheres, S. H. W. (2024). *Nature*, **628**, 450–457. 10.1038/s41586-024-07215-4 PMC11006616 38408488

[bb38] James, A. M. & Farnung, L. (2025). *Mol. Cell*, **85**, 1938–1951. 10.1016/j.molcel.2025.04.020 PMC12126155 40334658

[bb39] Karras, G. I., Kustatscher, G., Buhecha, H. R., Allen, M. D., Pugieux, C., Sait, F., Bycroft, M. & Ladurner, A. G. (2005). *EMBO J.* **24**, 1911–1920. 10.1038/sj.emboj.7600664 PMC1142602 15902274

[bb40] Kastner, B., Fischer, N., Golas, M. M., Sander, B., Dube, P., Boehringer, D., Hartmuth, K., Deckert, J., Hauer, F., Wolf, E., Uchtenhagen, H., Urlaub, H., Herzog, F., Peters, J. M., Poerschke, D., Lührmann, R. & Stark, H. (2008). *Nat. Methods*, **5**, 53–55. 10.1038/nmeth1139 18157137

[bb41] Kaur, U., Wu, H., Cheng, Y. & Narlikar, G. J. (2025). *Science*, **389**, eadr3831. 10.1126/science.adr3831 PMC12403922 40674492

[bb42] Knight, P. J., Thirumurugan, K., Xu, Y., Wang, F., Kalverda, A. P., Stafford, W. F., Sellers, J. R. & Peckham, M. (2005). *J. Biol. Chem.* **280**, 34702–34708. 10.1074/jbc.M504887200 16030012

[bb43] Kornberg, R. D. (1974). *Science*, **184**, 868–871. 10.1126/science.184.4139.868 4825889

[bb44] Langelier, M.-F., Billur, R., Sverzhinsky, A., Black, B. E. & Pascal, J. M. (2021). *Nat. Commun.* **12**, 6675. 10.1038/s41467-021-27043-8 PMC8602370 34795260

[bb45] Langelier, M.-F., Eisemann, T., Riccio, A. A. & Pascal, J. M. (2018). *Curr. Opin. Struct. Biol.* **53**, 187–198. 10.1016/j.sbi.2018.11.002 PMC6687463 30481609

[bb46] Larsson, A. (2014). *Bioinformatics*, **30**, 3276–3278. 10.1093/bioinformatics/btu531 PMC4221126 25095880

[bb47] Lehmann, L. C., Bacic, L., Hewitt, G., Brackmann, K., Sabantsev, A., Gaullier, G., Pytharopoulou, S., Degliesposti, G., Okkenhaug, H., Tan, S., Costa, A., Skehel, J. M., Boulton, S. J. & Deindl, S. (2020). *Cell. Rep.* **33**, 108529. 10.1016/j.celrep.2020.108529 PMC7116876 33357431

[bb48] Lehmann, L. C., Hewitt, G., Aibara, S., Leitner, A., Marklund, E., Maslen, S. L., Maturi, V., Chen, Y., van der Spoel, D., Skehel, J. M., Moustakas, A., Boulton, S. J. & Deindl, S. (2017). *Mol. Cell*, **68**, 847–859. 10.1016/j.molcel.2017.10.017 PMC5745148 29220652

[bb49] Leidecker, O., Bonfiglio, J. J., Colby, T., Zhang, Q., Atanassov, I., Zaja, R., Palazzo, L., Stockum, A., Ahel, I. & Matic, I. (2016). *Nat. Chem. Biol.* **12**, 998–1000. 10.1038/nchembio.2180 PMC5113755 27723750

[bb50] Li, L., Chen, K., Sia, Y., Hu, P., Ye, Y. & Chen, Z. (2024). *Nat. Struct. Mol. Biol.* **31**, 266–274. 10.1038/s41594-023-01174-6 38177688

[bb51] Li, M., Lahvic, J. L., Binder, V., Pugach, E. K., Riley, E. B., Tamplin, O. J., Panigrahy, D., Bowman, T. V., Barrett, F. G., Heffner, G. C., McKinney-Freeman, S., Schlaeger, T. M., Daley, G. Q., Zeldin, D. C. & Zon, L. I. (2019). *Nature*, **573**, E1. 10.1038/s41586-019-1489-4 31435017

[bb52] Liu, X., Li, M., Xia, X., Li, X. & Chen, Z. (2017). *Nature*, **544**, 440–445. 10.1038/nature22036 28424519

[bb53] Louder, R. K., Park, G., Ye, Z., Cha, J. S., Gardner, A. M., Lei, Q., Ranjan, A., Höllmüller, E., Stengel, F., Pugh, B. F. & Wu, C. (2024). *Cell*, **187**, 6849–6864. 10.1016/j.cell.2024.09.007 PMC11606799 39357520

[bb54] Lowary, P. T. & Widom, J. (1998). *J. Mol. Biol.* **276**, 19–42. 10.1006/jmbi.1997.1494 9514715

[bb55] Luger, K., Mäder, A. W., Richmond, R. K., Sargent, D. F. & Richmond, T. J. (1997). *Nature*, **389**, 251–260. 10.1038/38444 9305837

[bb56] Luzete-Monteiro, E. & Zaret, K. S. (2022). *Curr. Opin. Struct. Biol.* **75**, 102425. 10.1016/j.sbi.2022.102425 PMC9976633 35863165

[bb57] Makowski, M. M., Gaullier, G. & Luger, K. (2020). *J. Biosci.* **45**, 13. 31965991

[bb58] Malik, D., Deshmukh, A., Bilokapic, S. & Halic, M. (2025). *Cell Res.* **35**, 465–468. 10.1038/s41422-025-01103-w PMC12134373 40175616

[bb59] Markert, J. & Luger, K. (2021). *Trends Biochem. Sci.* **46**, 41–50. 10.1016/j.tibs.2020.08.010 32917506

[bb60] Markert, J., Zhou, K. & Luger, K. (2021). *Sci. Adv.* **7**, eabk2380. 10.1126/sciadv.abk2380 PMC8519567 34652950

[bb61] Matsumoto, S., Cavadini, S., Bunker, R. D., Grand, R. S., Potenza, A., Rabl, J., Yamamoto, J., Schenk, A. D., Schübeler, D., Iwai, S., Sugasawa, K., Kurumizaka, H. & Thomä, N. H. (2019). *Nature*, **571**, 79–84. 10.1038/s41586-019-1259-3 PMC6611726 31142837

[bb62] McGinty, R. K. & Tan, S. (2021). *Curr. Opin. Struct. Biol.* **71**, 16–26. 10.1016/j.sbi.2021.05.006 PMC8648869 34198054

[bb63] Mohapatra, J., Tashiro, K., Beckner, R. L., Sierra, J., Kilgore, J. A., Williams, N. S. & Liszczak, G. (2021). *eLife*, **10**, e71502. 10.7554/eLife.71502 PMC8683085 34874266

[bb64] Narlikar, G. J., Sundaramoorthy, R. & Owen-Hughes, T. (2013). *Cell*, **154**, 490–503. 10.1016/j.cell.2013.07.011 PMC3781322 23911317

[bb65] Ngo, T. T. M., Zhang, Q., Zhou, R., Yodh, J. G. & Ha, T. (2015). *Cell*, **160**, 1135–1144. 10.1016/j.cell.2015.02.001 PMC4409768 25768909

[bb66] Nodelman, I. M., Das, S., Faustino, A. M., Fried, S. D., Bowman, G. D. & Armache, J.-P. (2022). *Nat. Struct. Mol. Biol.* **29**, 121–129. 10.1038/s41594-021-00719-x PMC9107065 35173352

[bb67] Nodelman, I. M., Folkwein, H. J., Glime, W. S., Armache, J.-P. & Bowman, G. D. (2025). *Nat. Struct. Mol. Biol.* **32**, 1445–1455. 10.1038/s41594-025-01556-y PMC12879502 40437259

[bb68] Obaji, E., Haikarainen, T. & Lehtiö, L. (2018). *Nucleic Acids Res.* **46**, 12154–12165. 10.1093/nar/gky927 PMC6294510 30321391

[bb69] Obaji, E., Maksimainen, M. M., Galera-Prat, A. & Lehtiö, L. (2021). *Nat. Commun.* **12**, 3479. 10.1038/s41467-021-23800-x PMC8190142 34108479

[bb70] Osakabe, A., Takizawa, Y., Horikoshi, N., Hatazawa, S., Negishi, L., Sato, S., Berger, F., Kakutani, T. & Kurumizaka, H. (2024). *Nat. Commun.* **15**, 5187. 10.1038/s41467-024-49465-w PMC11239853 38992002

[bb71] Patel, A. B., Moore, C. M., Greber, B. J., Luo, J., Zukin, S. A., Ranish, J. & Nogales, E. (2019). *eLife*, **8**, e54449. 10.7554/eLife.54449 PMC6959994 31886770

[bb72] Pettersen, E. F., Goddard, T. D., Huang, C. C., Meng, E. C., Couch, G. S., Croll, T. I., Morris, J. H. & Ferrin, T. E. (2021). *Protein Sci.* **30**, 70–82. 10.1002/pro.3943 PMC7737788 32881101

[bb73] Pintilie, G., Zhang, K., Su, Z., Li, S., Schmid, M. F. & Chiu, W. (2020). *Nat. Methods*, **17**, 328–334. 10.1038/s41592-020-0731-1 PMC7446556 32042190

[bb74] Punjani, A. & Fleet, D. J. (2021). *J. Struct. Biol.* **213**, 107702. 10.1016/j.jsb.2021.107702 33582281

[bb75] Punjani, A., Rubinstein, J. L., Fleet, D. J. & Brubaker, M. A. (2017). *Nat. Methods*, **14**, 290–296. 10.1038/nmeth.4169 28165473

[bb76] R Core Team (2025). *R: A Language and Environment for Statistical Computing*. Vienna: R Foundation for Statistical Computing.

[bb77] Ren, M., Gut, F., Fan, Y., Ma, J., Shan, X., Yikilmazsoy, A., Likhodeeva, M., Hopfner, K.-P. & Zhou, C. (2024). *Nat. Commun.* **15**, 9407. 10.1038/s41467-024-53811-3 PMC11526172 39477986

[bb78] Ren, R., Ghassabi Kondalaji, S. & Bowman, G. D. (2019). *J. Biol. Chem.* **294**, 18181–18191. 10.1074/jbc.RA119.009782 PMC6885614 31636125

[bb79] Sellou, H., Lebeaupin, T., Chapuis, C., Smith, R., Hegele, A., Singh, H. R., Kozlowski, M., Bultmann, S., Ladurner, A. G., Timinszky, G. & Huet, S. (2016). *Mol. Biol. Cell*, **27**, 3791–3799. 10.1091/mbc.E16-05-0269 PMC5170603 27733626

[bb80] Sia, Y., Pan, H., Chen, K. & Chen, Z. (2025). *Science*, **388**, eadu5654. 10.1126/science.adu5654 40179160

[bb81] Singh, H. R., Nardozza, A. P., Möller, I. R., Knobloch, G., Kistemaker, H. A. V., Hassler, M., Harrer, N., Blessing, C., Eustermann, S., Kotthoff, C., Huet, S., Mueller-Planitz, F., Filippov, D. V., Timinszky, G., Rand, K. D. & Ladurner, A. G. (2017). *Mol. Cell*, **68**, 860–871.e7. 10.1016/j.molcel.2017.11.019 29220653

[bb82] Sondka, Z., Dhir, N. B., Carvalho-Silva, D., Jupe, S., Madhumita, McLaren, K., Starkey, M., Ward, S., Wilding, J., Ahmed, M., Argasinska, J., Beare, D., Chawla, M. S., Duke, S., Fasanella, I., Neogi, A. G., Haller, S., Hetenyi, B., Hodges, L., Holmes, A., Lyne, R., Maurel, T., Nair, S., Pedro, H., Sangrador-Vegas, A., Schuilenburg, H., Sheard, Z., Yong, S. Y. & Teague, J. (2024). *Nucleic Acids Res.* **52**, D1210–D1217. 10.1093/nar/gkad986 PMC10767972 38183204

[bb83] Sun, F.-H., Zhao, P., Zhang, N., Kong, L.-L., Wong, C. C. L. & Yun, C.-H. (2021). *Nat. Commun.* **12**, 1028. 10.1038/s41467-021-21302-4 PMC7884425 33589610

[bb84] Sundaramoorthy, R., Hughes, A. L., El-Mkami, H., Norman, D. G., Ferreira, H. & Owen-Hughes, T. (2018). *eLife*, **7**, e35720. 10.7554/eLife.35720 PMC6118821 30079888

[bb85] Suskiewicz, M. J., Zobel, F., Ogden, T. E. H., Fontana, P., Ariza, A., Yang, J., Zhu, K., Bracken, L., Hawthorne, W. J., Ahel, D., Neuhaus, D. & Ahel, I. (2020). *Nature*, **579**, 598–602. 10.1038/s41586-020-2013-6 PMC7104379 32028527

[bb86] Süveges, D., Gáspári, Z., Tóth, G. & Nyitray, L. (2009). *Proteins*, **74**, 905–916. 10.1002/prot.22183 18712826

[bb87] Tian, Y., Jia, Q., Li, M., Sia, Y., Hu, P., Chen, K., Li, M., Li, X., Xu, Z., Ma, L., Ye, Y., Lu, Y. & Chen, Z. (2025). *Life Metab.* **4**, loaf013. 10.1093/lifemeta/loaf013 PMC12125543 40453884

[bb88] Varadi, M., Anyango, S., Deshpande, M., Nair, S., Natassia, C., Yordanova, G., Yuan, D., Stroe, O., Wood, G., Laydon, A., Žídek, A., Green, T., Tunyasuvunakool, K., Petersen, S., Jumper, J., Clancy, E., Green, R., Vora, A., Lutfi, M., Figurnov, M., Cowie, A., Hobbs, N., Kohli, P., Kleywegt, G., Birney, E., Hassabis, D. & Velankar, S. (2022). *Nucleic Acids Res.* **50**, D439–D444. 10.1093/nar/gkab1061 PMC8728224 34791371

[bb89] Wagner, F. R., Dienemann, C., Wang, H., Stützer, A., Tegunov, D., Urlaub, H. & Cramer, P. (2020). *Nature*, **579**, 448–451. 10.1038/s41586-020-2088-0 PMC7093204 32188943

[bb90] Wang, L., Chen, K. & Chen, Z. (2021). *Nat. Commun.* **12**, 4057. 10.1038/s41467-021-24320-4 PMC8249414 34210977

[bb91] Weaver, T. M., Hoitsma, N. M., Spencer, J. J., Gakhar, L., Schnicker, N. J. & Freudenthal, B. D. (2022). *bioRxiv*, 2022.03.09.483662. 10.1038/s41467-022-33057-7 PMC9474862 36104361

[bb92] Willhoft, O., Ghoneim, M., Lin, C.-L., Chua, E. Y. D., Wilkinson, M., Chaban, Y., Ayala, R., McCormack, E. A., Ocloo, L., Rueda, D. S. & Wigley, D. B. (2018). *Science*, **362**, eaat7716.10.1126/science.aat771630309918

[bb93] Wolny, M., Batchelor, M., Bartlett, G. J., Baker, E. G., Kurzawa, M., Knight, P. J., Dougan, L., Woolfson, D. N., Paci, E. & Peckham, M. (2017). *Sci. Rep.* **7**, 44341. 10.1038/srep44341 PMC5347031 28287151

[bb94] Wolny, M., Batchelor, M., Knight, P. J., Paci, E., Dougan, L. & Peckham, M. (2014). *J. Biol. Chem.* **289**, 27825–27835. 10.1074/jbc.M114.585679 PMC4183817 25122759

[bb95] Wu, H., Muñoz, E. N., Hsieh, L. J., Chio, U. S., Gourdet, M. A., Narlikar, G. J. & Cheng, Y. (2023). *Science*, **381**, 319–324. 10.1126/science.adf4197 PMC10480058 37384669

[bb96] Yan, L., Wu, H., Li, X., Gao, N. & Chen, Z. (2019). *Nat. Struct. Mol. Biol.* **26**, 258–266. 10.1038/s41594-019-0199-9 30872815

[bb97] Ye, Y., Wu, H., Chen, K., Clapier, C. R., Verma, N., Zhang, W., Deng, H., Cairns, B. R., Gao, N. & Chen, Z. (2019). *Science*, **366**, 838–843. 10.1126/science.aay0033 PMC8442553 31672915

[bb98] Yuan, J., Chen, K., Zhang, W. & Chen, Z. (2022). *Nature*, **605**, 166–171. 10.1038/s41586-022-04658-5 35477757

[bb99] Zhang, H., Gu, Z., Zeng, Y. & Zhang, Y. (2024). *Structure*, **32**, 1222–1230. 10.1016/j.str.2024.05.013 38870940

[bb100] Zhang, M., Jungblut, A., Kunert, F., Hauptmann, L., Hoffmann, T., Kolesnikova, O., Metzner, F., Moldt, M., Weis, F., DiMaio, F., Hopfner, K.-P. & Eustermann, S. (2023). *Science*, **381**, 313–319. 10.1126/science.adf6287 37384673

[bb101] Zhong, E. D., Bepler, T., Berger, B. & Davis, J. H. (2021). *Nat. Methods*, **18**, 176–185. 10.1038/s41592-020-01049-4 PMC8183613 33542510

[bb102] Zhou, K., Gaullier, G. & Luger, K. (2019). *Nat. Struct. Mol. Biol.* **26**, 3–13. 10.1038/s41594-018-0166-x PMC7386248 30532059

[bb103] Zhu, F., Farnung, L., Kaasinen, E., Sahu, B., Yin, Y., Wei, B., Dodonova, S. O., Nitta, K. R., Morgunova, E., Taipale, M., Cramer, P. & Taipale, J. (2018). *Nature*, **562**, 76–81. 10.1038/s41586-018-0549-5 PMC6173309 30250250

