# Supplementary material for: Cryo-EM structure of ALC1 in an open conformation bound to a PARylated nucleosome
Source: Acta Crystallogr D Struct Biol. 2026 May 20;82(Pt 6):683–99. doi: 10.1107/S2059798326004158 (PMC13224931; doi:10.1107/S2059798326004158)
Supplement: Supplementary file 1 [file d-82-00683-sup1.pdf]

STRUCTURAL
BIOLOGY

Volume 82 (2026)

Supporting information for article:

Cryo-EM structure of ALC1 in an open conformation bound to a PARylated nucleosome

Hannah R. Bridges, Luka Bacic, Sebastian Deindl and Guillaume Gaullier

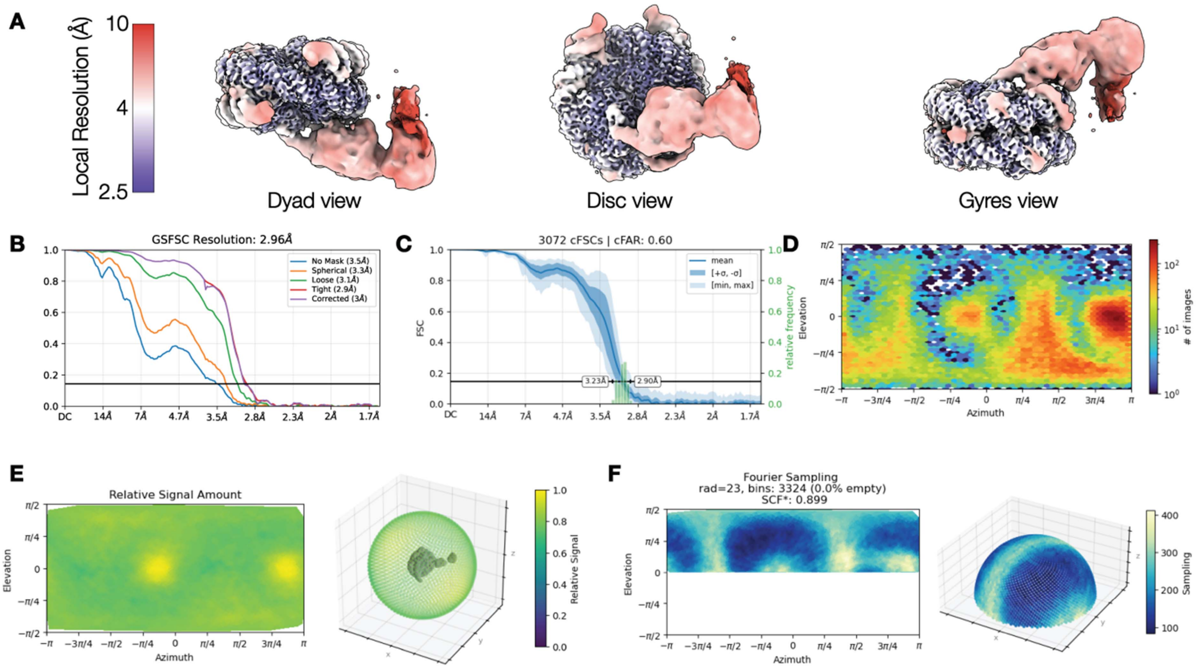

Figure S1 Validation of the cryo-EM map used to build the RLS with ModelAngelo. (A) Map colored by local resolution. (B) Gold-standard FSC curve. (C) Measure of resolution anisotropy by conical FSC area ratio (cFAR). (D) Euler angles distribution of the set of particles contributing to this reconstruction. (E) Relative signal versus viewing direction. (F) Distribution of Fourier sampling.

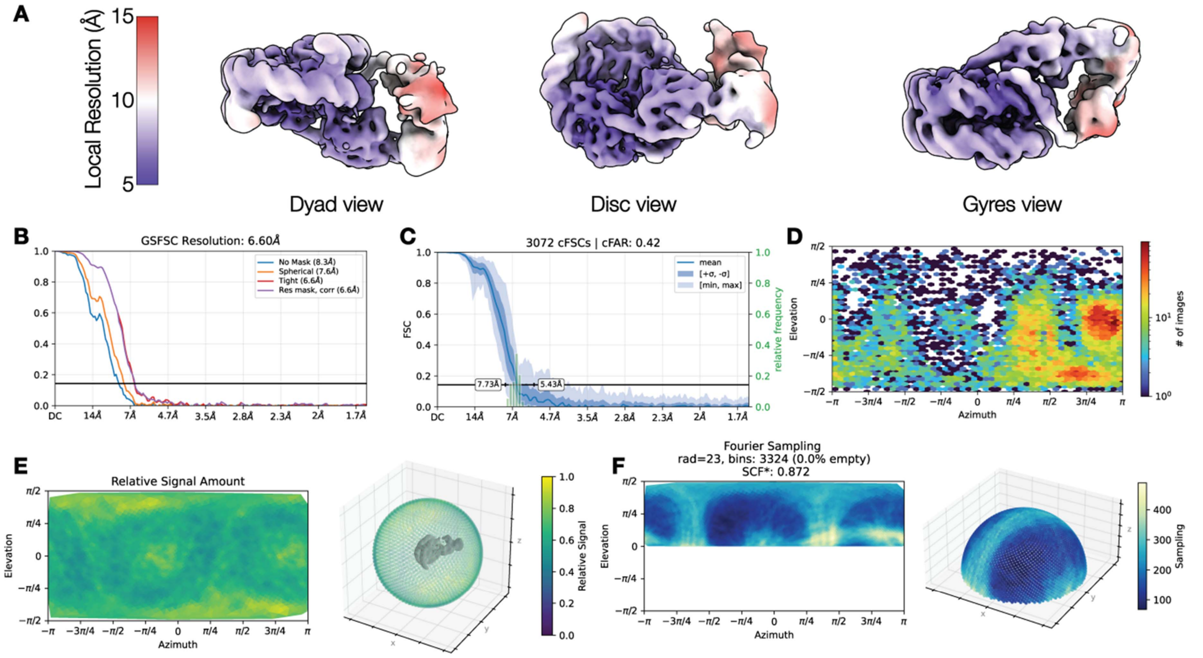

Figure S2 Validation of the main cryo-EM map. (A) Map colored by local resolution. (B) Gold-standard FSC curve. (C) Measure of resolution anisotropy by conical FSC area ratio (cFAR). (D) Euler angles distribution of the set of particles contributing to this reconstruction. (E) Relative signal versus viewing direction. (F) Distribution of Fourier sampling.

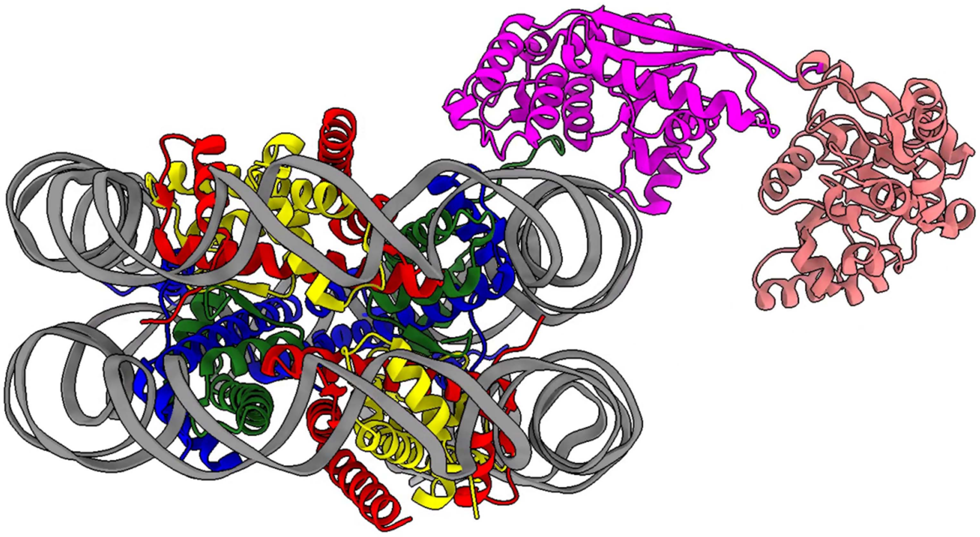

Figure S3 Movie of an interpolation between the closed and open conformations of nucleosome-bound ALC1. Closed conformation from PDB 7OTQ / EMD-13065, open conformation from this study (PDB 9T4V / EMD-55533).

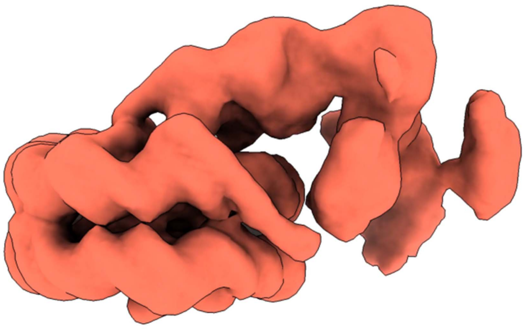

Figure S4 Movie of 3DVA (2).

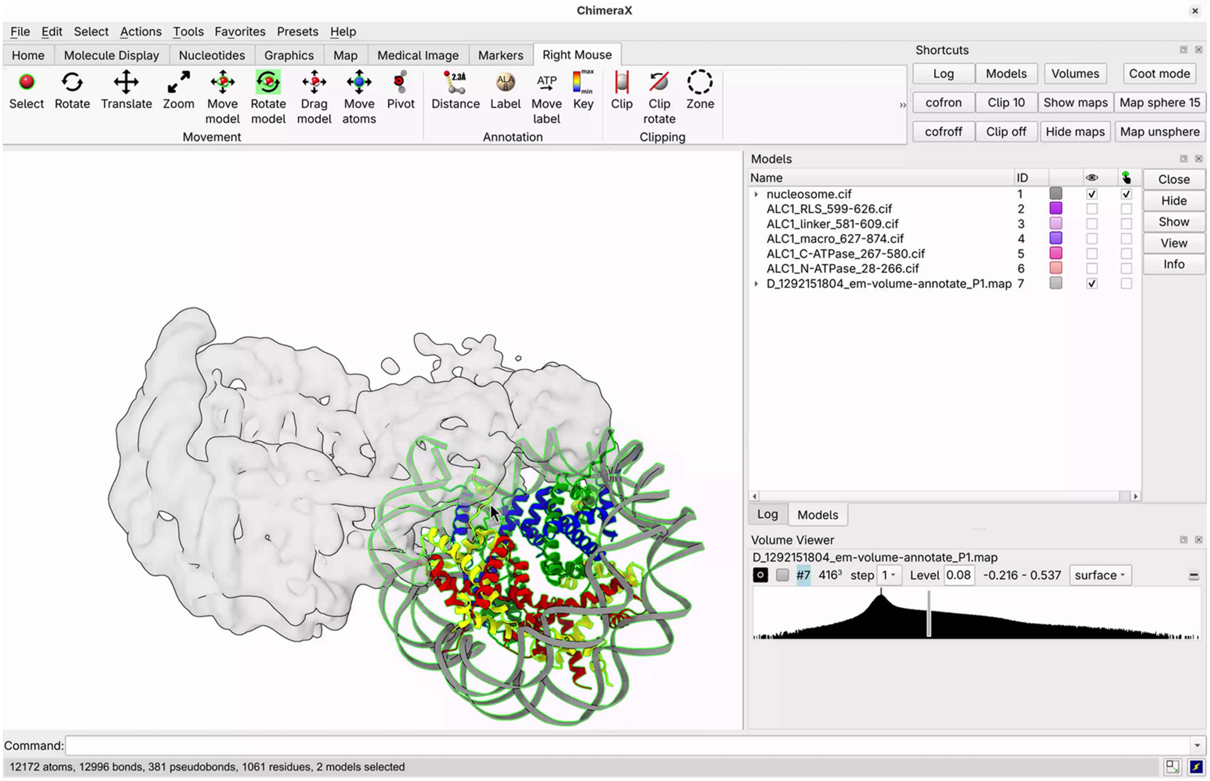

Figure S5 Movie of the rigid-body fitting procedure. Chains and domains follow the same color code as in Fig. 1 and Fig. 2. The movie was not sped up: the domain fitting procedure did take less than 5 minutes. Some of the residues of ALC1 following each other in sequence but that ended up in different fragments for the domain fitting procedure are shown as spheres: V266 (in the N-ATPase fragment) and A267 (in the C-ATPase fragment) are colored in green; Y580 (in the C-ATPase fragment) and S581 (in the linker fragment) are colored in cyan. The placement of the linker helix, C-ATPase lobe and N-ATPase lobe resulted in these sequence-adjacent residues being brought close to each other in space, which gave additional confidence in the rigid-body fitting solutions.

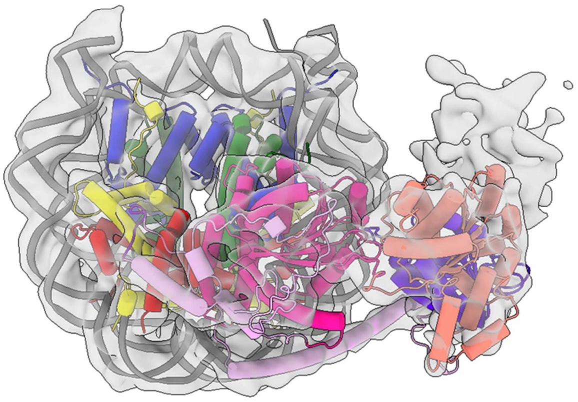

Figure S6 Movie of the flexible fitting procedure. Chains and domains follow the same color code as in Fig. 1 and Fig. 2.. The movie shows a morph (interpolation over 12 steps) between the initial model and the final, refined model.

Table S1 Statistics from data collection, image processing, model building and validation.

ND: not determined.

**Data collection**	Same information as in Table 1 in (Bacic et al., 2021)
Acceleration voltage (kV)	300
Spherical aberration (mm)	2.7
Image pixel size (Å/pixel)	0.84
Total electron exposure (e-/Å2)	45
Number of movie frames	40
Electron exposure per frame (e-/Å2)	1.125
Nominal defocus range (µm)	–1.0 to –2.5
Number of exposures	33 998

collected		
Number of exposures used (Bacic et al., 2021)	26 747	
Number of exposures used (this re-analysis)	28 448	
**Image processing**	**RLS resolved (NU Refinement 3)**	**ALC1 activation intermediate (Local Refinement 2)**
Number of micrographs used for picking	28 448	28 448
Number of picked particles	2 001 174	2 001 174
Number of particles used for reconstruction	57 036	15 740
Point group symmetry imposed	C1	C1
Conical FSC area ratio (cFAR)	0.60	0.42
Fourier sampling compensation factor (SCF*)	0.899	0.872
Map sharpening B-factor (Å^2)	-24.3	-300
Global resolution at FSC = 0.143 (Å)	2.96	6.60
**EMDB accession code**	EMD-55534	EMD-55533
**Model building and refinement**	**RLS resolved (NU Refinement 3)**	**ALC1 in open conformation (Local Refinement 2)**
Initial models	Output of ModelAngelo run with sequence information.	Nucleosome from PDB 8B0A.<br>ALC1 residues 599-626 from the output of ModelAngelo run on the map from NU Refinement 3 with sequence information.<br>Other ALC1 domains from AlphaFold-DB AF-Q86WJ1-F1-v3.
Number of protein	796	1610

residues		
Number of protein atoms (including hydrogen atoms)	6 320 (ND)	12 805 (25 971)
Number of DNA residues	312	298
Number of DNA atoms (including hydrogen atoms)	6 377 (ND)	6 109 (9 454)
Number of ligands	0	0
Number of ions	0	0
Number of water molecules	0	0
**Model validation**	**RLS resolved (NU Refinement 3)**	**ALC1 activation intermediate (Local Refinement 2)**
Protein B-factors (min/median/max, Å^2)	ND	14 / 306 / 903
DNA B-factors (min/median/max, Å^2)	ND	67.3 / 319 / 1030
Bond lengths RMSD (Å)	ND	0.008
Bond angles RMSD (º)	ND	1.292
MolProbity score	ND	1.75
Clashscore	ND	7.48
Rotamer outliers (%)	ND	0.73
C-beta deviations	ND	0
Ramachandran favored (%)	ND	95.16
Ramachandran allowed (%)	ND	3.52
Ramachandran outliers (%)	ND	1.32
**Model to map fit**	**RLS resolved (NU Refinement 3)**	**ALC1 activation intermediate (Local Refinement 2)**

CC volume	ND	0.7191
CC mask	ND	0.7439
CC peaks	ND	0.5931
Protein Q-scores (min/median/max)	ND	-0.845 / 0.282 / 0.847
DNA Q-scores (min/median/max)	ND	-0.610 / 0.398 / 0.805
**PDB accession code**	Not deposited	pdb_00009T4V
